# Caspase-dependent cell death-associated release of nucleosome and damage-associated molecular patterns

**DOI:** 10.1038/cddis.2014.450

**Published:** 2014-10-30

**Authors:** S Yoon, S J Park, J H Han, J H Kang, J-h Kim, J Lee, S Park, H-J Shin, K Kim, M Yun, Y-J Chwae

**Affiliations:** 1 Department of Microbiology, Ajou University School of Medicine, Suwon, Korea; 2 Department of Biomedical Sciences, Ajou University, Suwon, Korea; 3 Department of Pathology, Ajou University School of Medicine, Suwon, Korea; 4 Electron Microscopy Laboratory, Eulji University, Seongnam, Korea; 5 Bio-Medical Science Co. Ltd, Seoul, Korea; 6 Department of Nuclear Medicine, College of Medicine, Yonsei University, Seoul, Korea

## Abstract

Apoptosis, which is anti-inflammatory, and necrosis, which is pro-inflammatory, represent the extremes of the cell death spectrum. Cell death is complex and both apoptosis and necrosis can be observed in the same cells or tissues. Here, we introduce a novel combined mode of cellular demise – caspase-dependent regulated necrosis. Most importantly, it is mainly characterized with release of marked amount of oligo- or poly-nucleosomes and their attached damage-associated molecular patterns (DAMPs) and initiated by caspase activation. Caspase-activated DNase has dual roles in nucleosomal release as it can degrade extracellularly released chromatin into poly- or oligo-nucleosomes although it prohibits release of nucleosomes. In addition, osmotically triggered water movement following Cl^−^ influx and subsequent Na^+^ influx appears to be the major driving force for nucleosomal and DAMPs release. Finally, Ca^2+^-activated cysteine protease, calpain, is an another essential factor in nucleosomal and DAMPs release because of complete reversion to apoptotic morphology from necrotic one and blockade of nucleosomal and DAMPs release by its inhibition.

Apoptosis is characterized by membrane blebbing, cellular shrinkage, nuclear condensation, nuclear fragmentations, oligo-nucleosomal DNA fragmentation and formation of apoptotic bodies. These characteristics are attributed mainly to the caspase family of cysteine proteases.^1,2^ Necrosis is distinguished from apoptosis by cellular swelling, plasma membrane rupture, absence of oligo-nucleosomal degradation and, finally, rapid lysis of cells and cellular constituents including damage-associated molecular patterns (DAMPs) are massively exuded extracellularly to activate inflammatory and immune responses. ^3, 4, 5^

Calpains are a family of Ca^2+^-activated cysteine proteases consisting of 15 genes. Among them, *μ*-calpain (calpain I) and m-calpain (calpain II) are ubiquitously expressed in most cells as a heterodimer consisting of a large subunit (80 kDa; calpain 1 of *μ*-calpain and calpain 2 of m-calpain) and a common small subunit (29 kDa; calpain S1), which is processed into a smaller heterodimer (18–78 kDa) upon activation by Ca^2+^. Ubiquitous calpains are regulated by an endogenous inhibitor, calpastatin.^6^

It has long been observed that both apoptosis and necrosis can be simultaneously detected in tissues or cell culture. Therefore, apoptosis and necrosis have been assumed to be two extremes of the cell death spectrum capable of inter-conversion by key regulators.^5,7^ In this study, we introduce a novel mode of cell death involving the combination of apoptosis and necrosis, being a caspase-dependent process with necrotic morphology, involving the active release of DAMPs bound to nucleosomes.

## Results

### Release of nucleosomes and DAMPs from amino-acid-deprived HeLa cells

**Figure 1:**
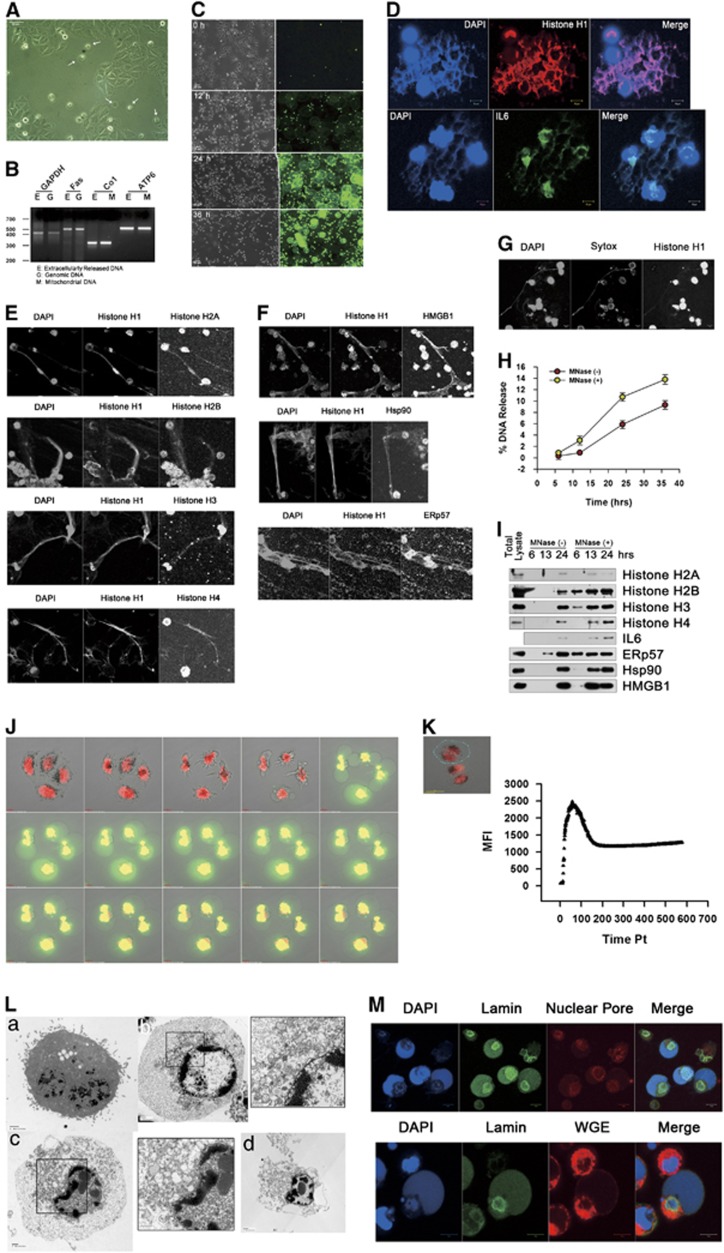
Release of nucleosomes and DAMPs from amino-acid-depleted HeLa cells. (**A**) An inverted microscopic image of HeLa cells in the condition of amino-acid depletion. Arrows designate dying HeLa cells. (**B**) Genomic sequences of glyceraldehyde-3-phosphate dehydrogenase (*GAPDH*), Fas, cytochrome oxidase subunit 1 (*Co1*) and ATP synthase subunit 6 (*ATP6*) were PCR amplified from extracellularly released DNA, genomic or mitochondrial DNAs. (**C**) Inverted and fluorescent microscopic images were taken from amino-acid-deprived HeLa cells in the presence of SYTOX, a membrane-impermeable DNA dye. HeLa cells deprived of amino acids for 15 h were fluorescence stained with histone H1 or IL6 antibodies (**D**), histone H2A, H2B, H3 or H4 antibodies (**E**) or HMGB1, Hsp90 or ERp57 antibodies (**F**) in combination with histone H1 antibody and 4',6-diamidino-2-phenylindole (DAPI). (**G**) Amino-acid-deprived HeLa cells were stained with SYTOX to determine viability, fixed and stained with DAPI and histone H1 antibodies. (**H**) Amino-acid-deprived HeLa cells were untreated or treated with MNase (500 mU/ml) for 10 min. Released DNA was quantitated at the indicated times. Data from triplicate samples are presented as mean±S.D. (**I**) Conditioned media from amino-acid-deprived HeLa cells treated or untreated with MNase were western blotted with histone H1, 2B, H3, H4, IL6, ERp57, HMGB1 or Hsp90 antibodies. (**J**) Images captured every hour from live imaging of amino-acid-deprived HeLa cells with SYTOX (green) and DRAQ5, membrane-permeable DNA dye (red). (**K**) SYTOX fluorescent intensities were measured from circularized areas of live imaging of amino-acid-deprived cells in 5-min intervals. (**L**) TEM images of control cells (**L**a) and amino-acid-deprived HeLa cells (**L**b–**L**d). (**M**) Amino-acid-deprived HeLa cells were fluorescence stained with lamin and nuclear pore antibodies, or lamin antibody and wheat germ agglutinin (WGE)

Amino-acid-deprived HeLa cells die and release of various pro-inflammatory mediators,^8,9^ with cellular morphology displaying detached and round plasma membranes, and diminished nuclei (Figure 1A). Surprisingly, when loaded with membrane-impermeable DNA dye, SYTOX Green, extracellular DNA release was revealed (Figure 1C). Amplification of genomic and mitochondrial gene sequences from the released DNA indicated the DNA was originated from nuclei and mitochondria (Figure 1B). Confocal microscopy revealed the released DNA co-stained with all major histones (Figure 1E), could be stained with SYTOX analogous with cellular bodies (Figure 1G), demonstrating the release of nucleosomes by dying cells. DNA also colocalized with interleukin 6 (IL6), high mobility group protein B1 (HMGB1), heat shock protein (Hsp) 90 and ERp57, a thiol oxidoreductase of the endoplasmic reticulum, which are characterized DAMPs (Figures 1D and F). The released DNAs and DAMPs was significantly increased by partial digestion with micrococcal nuclease (MNase) (Figures 1H and I), indicative of connection of released nucleosomes and DAMPs to cellular bodies. In live imaging, cells loaded with SYTOX Green and membrane-permeable DNA dye, DRAQ5, showed typical apoptotic morphologies, such as vigorous nuclear and cytoplasmic shrinkage, at early times, with abrupt plasma membrane swelling and the release of genomic DNA being evident after 5 h. DNA release continued slowly over the next 5 h together with slow contractions of swelled plasma membrane (Figures 1J and K,Supplementary Movie 1). Transmission electron microscopy (TEM) demonstrated swelled but intact nuclear membrane, partially condensed chromatin in the vicinity of nuclear membrane, sparse chromatin within the nucleoplasm and chromatin-like electron-dense material in the cytosol. Later examination of cells revealed disintegrated plasma membranes and small deflated nuclei with intact nuclear membranes (Figure 1L). Confocal microscopy revealed nuclei delineated by lamin A/C and nuclear pores appeared to have little DNA. In contrast, almost all DNA was located in the cytoplasm (Figure 1M), indicating that the cytosolic, chromatin-like, electron-dense material evident in TEM was definitely chromatin.

### Apoptotic features of dying HeLa cells during amino-acid deprivation

During nucleosomal release (Supplementary Figure 1a, upper left), cell death measured by propidium iodide (PI) staining increased over time (Supplementary Figure 1a, upper middle), and mitochondrial membrane potential rapidly decreased (Supplementary Figure 1a, upper left). Interestingly, the increased activity of caspases 3 and 7 noted until 24 h showed similar time courses with DNA release (Supplementary Figure 1a, lower left). In contrast, cells did not show any Annexin V-positive populations (Supplementary Figure 1a, lower middle) and TUNEL-positive populations (Supplementary Figure 1a, lower right) in accordance with no subG1 population (Supplementary Figure 1b). The cells showed cleavage of effector caspases and degradation of caspase substrates (Supplementary Figure 1c). Collectively, cells showed several apoptotic features, albeit no DNA fragmentation and no exposure of phosphatidylserine.

### Nucleosomal release is a common phenomenon provoked by various cytotoxic stimuli from various cells

**Figure 2:**
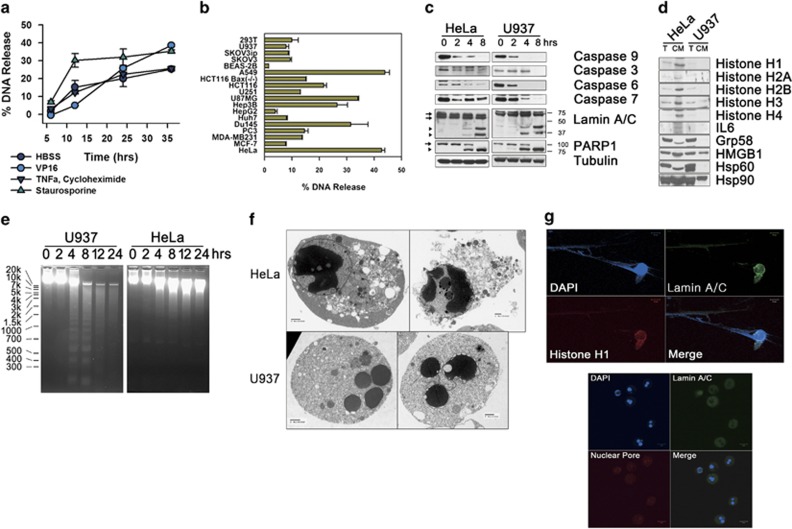
Nucleosome release is a common phenomenon provoked by various cytotoxic stimuli from various cells. (**a**) Death of HeLa cells was caused by incubation with amino-acid-depleted medium (*HBSS*), VP16 (100 *μ*M), TNF-*α* (50 ng/ml) plus cycloheximide (25 *μ*g/ml), or staurosporine (1 *μ*g/ml). Released DNA was measured with PicoGreen DNA dye. (**b**) Various human cell lines were incubated with staurosporine (1 *μ*g/ml) to the time when >50% of cellular viability was reduced in a Calcein assay to detect extracellularly released DNA. (**c**) Staurosporine-treated HeLa cells and U937 cells were western blotted with caspases 9, 3, 6, 7, lamin A/C, PARP-1 and tubulin antibodies. Arrows and arrow heads designate parental proteins and cleaved fragments, respectively. (**d**) Total lysates (*T*) and conditioned medium (*CM*) from staurosporine-treated HeLa and U937 cells were western blotted with histone (H1, H2A, H2B, H3, H4), IL6, ERp57, HMGB1, Hsp60 and Hsp90 antibodies. (**e**) Genomic DNAs prepared from staurosporine-treated HeLa and U937 cells were separated by agarose gel electrophoresis. (**f**) Electron microscopy images were taken from HeLa and U937 cells treated with staurosporine for 8 h. (**g**) HeLa cells (upper panel) and U937 cells (lower panel) treated with staurosporine for 7 h, were fluorescence-stained with lamin A/C antibody, nuclear pore antibody and DAPI. Data from triplicate samples are presented as mean±S.D. (**a** and **b**)

Nucleosomal and DAMPs release is not a phenomenon confined to amino-acid deprivation. As well-known apoptosis inducers including an anticancer drug, VP16, tumor necrosis factor-alpha (TNF*α*) plus cycloheximide or staurosporine could promote the release of DNA in HeLa cells (Figure 2a). Several human cell lines showed comparable or lesser DNA release with HeLa when staurosporine treated until >50% of cellular viabilities reduced (Figure 2b) indicating that nucleosomal release is a phenomenon occurred by various cytotoxic stimuli formerly known to induce apoptosis, regardless of its cellular specificity. For example, U937 displayed activated caspases, cleavage of poly ADP ribose polymerase-1 (PARP-1) and lamin A/C (Figure 2c), but little or no release of nucleosomes and DAMPs (Figures 2b and d) with definitive DNA laddering and nuclear fragmentations, whereas HeLa did not show any DNA and nuclear fragmentation in both staurosporine treatment (Figures 2e and f) and amino-acid deprivation (Figure 1I and Supplementary Figure 1a, lower right and 1b). In confocal microscopy, staurosporine treatment showed nucleosomal release in HeLa, in contrast, nuclear fragmentations in U937 (Figures 1D–F and 2g), suggesting apoptotic cell death without fragmentation of intracellular DNA and nuclei would be a key figure of the cell death releasing nucleosomes and DAMPs.

### Release of nucleosomes and DAMPs is not associated with NETosis

Morphological features of dying HeLa appear to be very similar with NETosis, in that cells release DAMPs attached to nucleosomes instead of antimicrobial molecules. Regulatory factors of NETosis include Nox-derived reactive oxygen species (ROS), autophagy and histone citrullination by peptidylarginine deaminase (PAD).^10^ Autophagy, ROS and PAD did not confer any effect on nucleosomal and DAMPs release investigated through specific inhibitors and short hairpin RNA (shRNA) transfection (Supplementary Figures 2). Therefore, cell death releasing nucleosomes and NETosis are totally different from one another, although morphologically very similar.

### Comparison between cell death involving release of nucleosomes and DAMPs, and primary necrosis

**Figure 3:**
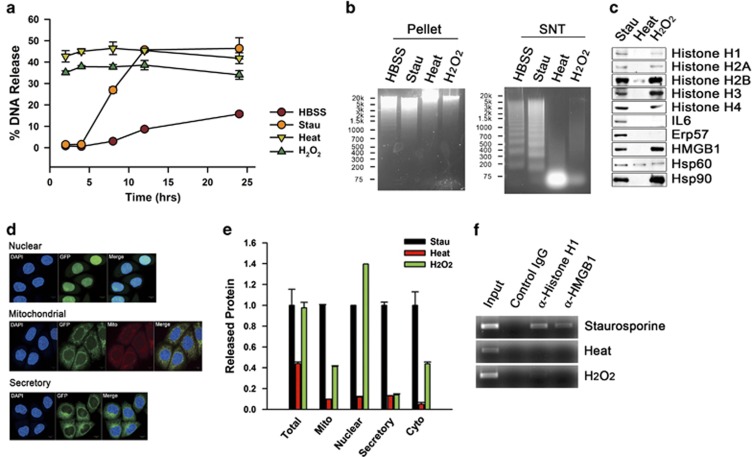
Comparison between cell death involving release of nucleosomes and DAMPs, and primary necrosis. HeLa cells were depleted of amino acids (*HBSS*), treated with staurosporine (1 *μ*g/ml) (*Stau*) or H_2_O_2_ (32 mM) (*H*_*2*_*O*_*2*_), or heated at 56 °C for 1 h and incubated at 37 °C (*Heat*) during the indicated time periods. DNA release was measured with PicoGreen and data from triplicate samples are presented as mean±S.D. (**a**). Cellular genomic DNA (*Pellet*) and released DNA (*SNT*) were separated in agarose gels with molecular weight marker from the cells with amino-acid deprivation for 24 h, staurosporine treatment for 12 h, heating at 56 °C for 1 h and incubation at 37 °C for 3 h, or H_2_O_2_ treatment for 4 h (**b**). Released protein was western blotted for histones, IL6, ERp57, HMGB1, Hsp60 or Hsp90 (**c**). HeLa cells expressing green fluorescence protein (GFP) targeted to nucleus, mitochondria or secretory pathway were imaged via confocal microscopy with DAPI (**d**). Released GFP (*Mito, Nuclear, Secretory*) from HeLa cells was measured with GFP ELISA, total released protein (*Total*) was measured by the Bradford assay, and released lactate dehydrogenase (LDH; *Cyto*) was measured by a LDH assay (**e**). Data executed as triplicate are presented as mean values of released protein relative to released protein from staurosporine-treated HeLa cells±S.D. The concentrated conditioned medium from HeLa cells was immunoprecipitated with control IgG, anti-histone H1 antibody, or anti-HMGB1 antibody. The purified DNA from the antibody–protein–DNA complexes was PCR-amplified for *GAPDH* sequences (**f**)

Next, we compared the cell death with primary necrosis. Primary necrosis induced by heating or H_2_O_2_ treatment showed earlier peak of DNA release than amino-acid deprivation or staurosporine treatment (Figure 3a). No fragmentation of genomic DNA was evident in all the conditions (Figure 3b, left panel). The released DNA from heated or H_2_O_2_-treated cells were completely degraded, whereas, surprisingly, the DNA released from staurosporine-treated and amino-acid-depleted cells showed classical apoptotic DNA cleavages into inter-nucleosomal fragments of roughly 180-base pairs and their multiples (Figure 3b, right panel). Heated cells released few histones and DAMPs. On the contrary, H_2_O_2_-treated cells released histones and DAMPs (Figure 3c). Total protein released from staurosporine-treated cells was analogous with that from H_2_O_2_-treated cells, but much more than from heated cells. The amount of released mitochondria, secretory pathway and cytosol proteins of staurosporine-treated cells was markedly higher than those of heated or H_2_O_2_-treated cells, investigated by GFP ELISA with conditioned media from cells expressing mitochondria-targeted GFP or GFP of secretory pathway, or LDH assay for detecting release of cytosolic components (Figures 3d and e). Furthermore, histone H1 and HMGB1 released from staurosporine-treated cells were bound to the released DNA, although those from heated or H_2_O_2_-treated cells were not, according to chromatin immunoprecipitation (ChIP) assay with conditioned medium (Figure 3f), implying that the cell death induced by staurosporine treatment releases proteins containing higher mitochondrial, cytosolic and secretory components, and DNAs as poly- or oligo-nucleosomes in comparison with primary necrosis in which totally degraded DNA and disjoined histones and DAMPs from DNA are released from the dying cells.

### Release of nucleosomes and DAMPs in dying HeLa cells is dependent on caspase

**Figure 4:**
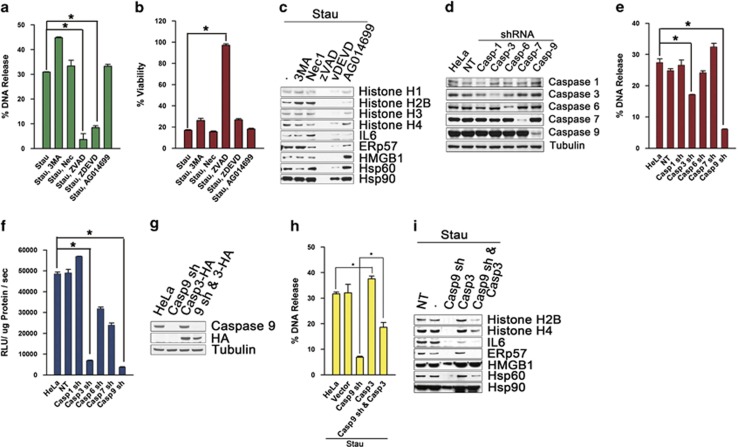
Release of nucleosomes and DAMPs from dying HeLa cells is dependent on caspase. HeLa cells were incubated with staurosporine (1 *μ*g/ml) in the presence of 3MA (10 mM), necrostatin-1 (*Nec*, 20 *μ*M), zVAD-fmk (20 *μ*M), zDEVD-fmk (20 *μ*M) or AG014699 (10 *μ*M) for 8 h. DNA release (**a**) and % viability (**b**) were measured by PicoGreen DNA dye method and Calcein assay, respectively. Released protein was western blotted for histones, IL6, ERp57, Hsp60 and Hsp90 (**c**). HeLa cells knocked-down by shRNA for caspases 1, 3, 6, 7 and 9 were confirmed on decreases of caspases by western blots (**d**), incubated with staurosporine, and DNA release at 8 h (**e**), and caspases 3 and 7 activity at 6 h (**f**) were monitored. HeLa cells knocked-down caspase 9 and/or overexpressing HA-tagged caspase 3 (**g**) were staurosporine-treated for 8 h, where released DNAs were measured by PicoGreen method (**h**) and released protein was detected by Western blot for histone H2B, histone H4, IL6, ERp57, HMGB1, Hsp60 or Hsp90 (**i**). Data from triplicate samples are presented as mean±S.D. (**a**, **b**, **e**, **f** and **h**). *P value<0.01

To explore molecular mechanisms of cell death, the effects of cell death inhibitors were examined. There were no effects on release of nucleosome and DAMPs with co-treatment of Necrostatin-1, an inhibitor of necroptosis, or AG14699, an inhibitor of PARP-1-dependent cell death. Co-treatment with 3-methyl adenine (3MA), an inhibitor of autophagy, increased DNA release as well as protein release even further. On the contrary, nucleosomal and DAMPs releases were strikingly reduced by co-treatment with pan-caspase inhibitor zVAD-fmk and caspase 3 inhibitor zDEVD-fmk in staurosporine-treated and amino-acid-deprived cells (Figures 4a and c, and Supplementary Figures 5a and c). In addition, cellular viability was increased markedly using zVAD-fmk co-treatments with staurosporine, but not in the condition of amino-acid depletion (Figure 4b and Supplementary Figure 5b). In cells transfected with shRNA for caspases 1, 3, 6, 7 and 9 (Figure 4d), knock-down of caspases 3 or 9 significantly blocked DNA release (Figure 4e and Supplementary Figure 5d), which also showed notably decreased effector caspase activities (Figure 4f and Supplementary Figure 5e). Cells overexpressing caspase 3 displayed slight increase in DNA release than controls. Moreover, increased DNA and protein release were evident in cells overexpressing caspase 3 but knocked-down for caspase 9 when compared with cells knocked-down for caspase 9 (Figures 4g–i), indicating that caspases 3 and 9 are essential in release of nucleosomes and DAMPs.

### Roles of caspase-activated DNase in nucleosomal release in dying HeLa cells

**Figure 5:**
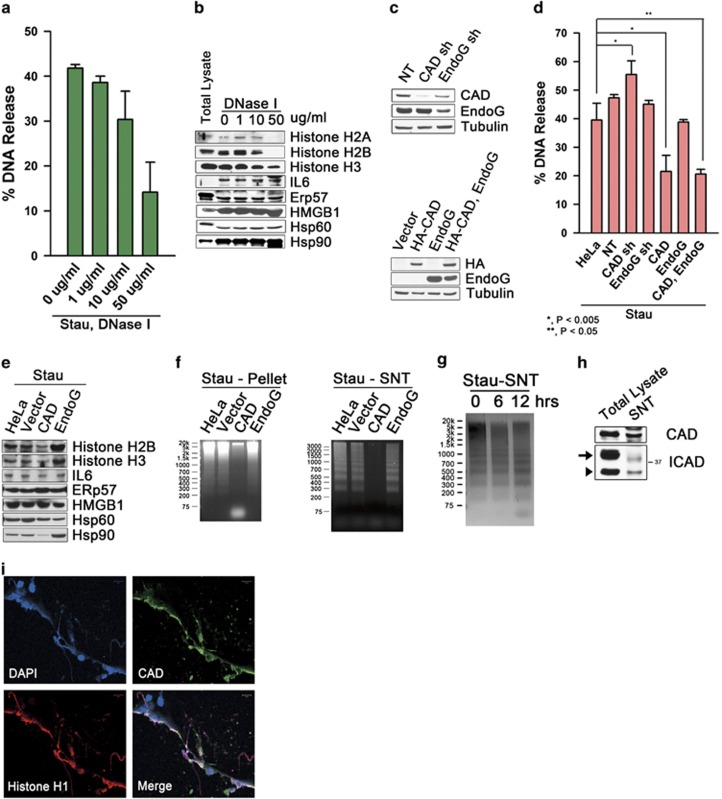
Roles of CAD in release of nucleosomes and DAMPs from dying HeLa cells. Death of HeLa cells was induced by treatment of staurosporine (1 *μ*g/ml) together with DNase I of the indicated concentrations, and released DNA (**a**), or histones and DAMPs (**b**) were identified by PicoGreen staining or western blot, respectively. In HeLa cells, either knocked-down or overexpressed for CAD or EndoG, confirmed by western blots (**c**), release of DNA (**d**) or histones and DAMPs (**e**) were measured by PicoGreen staining or western blots, respectively, after staurosporine treatments. (**f**) HeLa cells or cells transfected with control vector, CAD cDNA or EndoG cDNA were incubated with staurosporine for 10 h, from which genomic DNA (left panel) or released DNA (right panel) was separated by agarose gel electrophoresis. (**g**) Released DNA from HeLa cells treated with staurosporine for 10 h, was further incubated for the indicated times and analyzed by agarose gel electrophoresis. (**h**) CAD and ICAD were identified by western blot at the conditioned medium from staurosporine-treated HeLa cells (arrow: large isoform of ICAD; arrow head: small isoform of ICAD). (**i**) HeLa cells were incubated in staurosporine for 7 h and fluorescent-stained for CAD and histone H1. Data executed from triplicate samples are presented as mean±S.D. (**a** and **d**)

An intriguing finding was that apoptotic DNA laddering was observed only in the released DNA but not in nuclear DNA (Figure 3b). This prompted investigation of the role of caspase-activated DNase (DFF40/CAD) and its mitochondrial equivalent, endonuclease G (EndoG), which both function in DNA fragmentation during apoptosis. Addition of DNase I extracellularly during cell death decreased DNA release (Figure 5a) and release of histones (Figure 5b) in dose-dependent manners, implying that DNase activity impaired nucleosomal release. Moreover, knock-down of CAD increased DNA release and overexpression of CAD decreased release of DNA and histones, whereas there was no effect on release of DAMPs except for Hsp90, although knock-down or overexpression of EndoG had no effect on release of both nucleosomes and DAMPs (Figures 5c–e). Overexpression of CAD induced complete degradation of genomic and released DNAs (Figure 5f). These data are suggesting that CAD inhibits nucleosomal release through degrading nuclear DNA even if implicated in fragmentation of released nucleosomes, possibly in the extracellular space. Supporting this notion, released DNAs were additionally fragmentized by further *in vitro* incubation (Figure 5g), and CAD as well as inhibitor of CAD (ICAD) were released bound to extracellular released nucleosomes (Figures 5h and i).

### Chloride channels are associated with release of nucleosome and DAMPs

**Figure 6:**
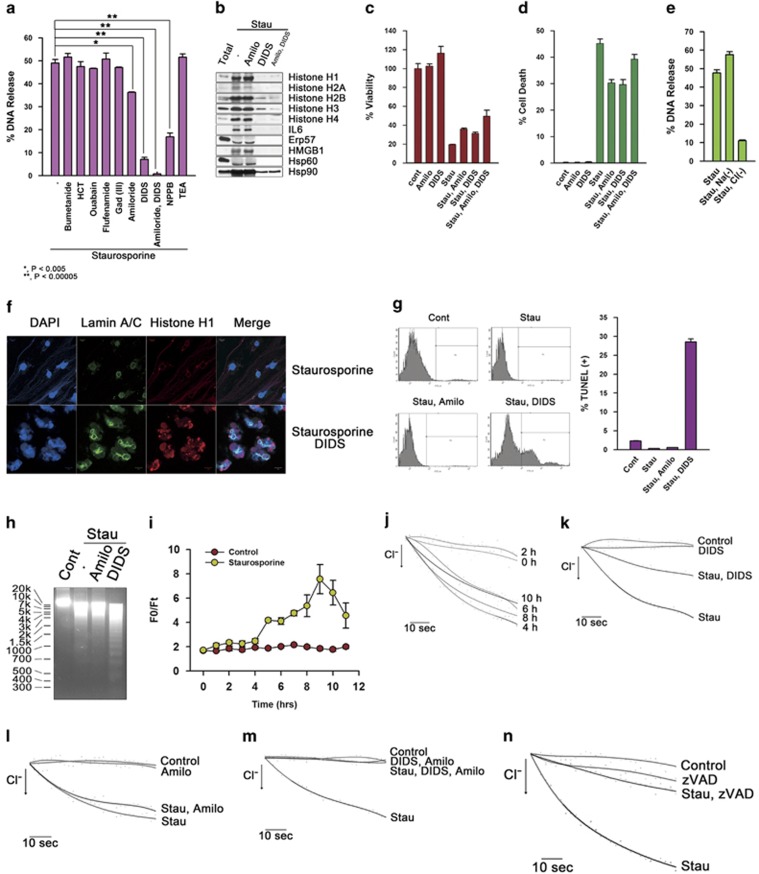
Chloride channels are associated with the release of nucleosomes and DAMPs in dying HeLa cells. (**a**) HeLa cells were incubated with staurosporine (1 *μ*g/ml) in combination with various ion channel inhibitors for 12 h; an inhibitor of epithelial Na^+^ channel, amiloride hydrochloride hydrate; Cl^−^ channel inhibitors, DIDS (200 *μ*M) and NPPB (200 *μ*M); an inhibitor of Na^+^/Cl^−^ cotransporter, HCT (100 *μ*M); an inhibitor of Na^+^ K^+^ ATPase, ouabain (100 *μ*M); an inhibitor of non-selective cation channel, flufenamide (100 *μ*M); an inhibitor of stretch-activated ion channel, Gad (III) chloride hexahydrate (40 *μ*M); an inhibitor of K^+^ channel, tetraethyl ammonium chloride (TEA) (5 mM); an inhibitor of Na^+^/K^+^ cotransporter, bumetanide (100 *μ*M). The released DNA was measured with PicoGreen dye (**a**) and the released protein from cells treated with staurosporine together with solvent, amiloride, DIDS or amiloride and DIDS was western blotted for histones and DAMPs (**b**). HeLa cells were treated with solvent or ion channel inhibitors with or without staurosporine for 8 h. Viability was measured with Calcein assay (**c**) and cell death was detected by SYTOX red staining (**d**). HeLa cells were incubated in MEM, Na^+^-deficient medium or Cl^−^-deficient medium with staurosporine (1 *μ*g/ml) for 10 h, and the released DNA was measured by PicoGreen (**e**). HeLa cells were incubated with staurosporine in combination with control solvent or DIDS. Nuclear fragmentation was detected with confocal microscopy (**f**), TUNEL staining (**g**) or agarose gel electrophoresis after genomic DNA preparation (**h**). Cellular chloride ion contents were measured by MQAE fluorescence in cells incubated with MEM containing either control solvent or staurosporine at the indicated time periods (**i**). Cellular Cl^−^ currents were measured in cells treated with staurosporine by halide-sensitive YFP quenching at the indicated times (**j**). HeLa cells treated with solvent or staurosporine in the presence or absence of DIDS, amiloride, DIDS and amiloride, or zVAD for 4 h and chloride ion currents were measured with halide-sensitive YFP quenching methods (**k**–**n**). Data performed in triplicate are presented as mean±S.D. (**a**, **c**, **d** and **e**, right panel of **g** and **i**)

Release of genomic DNA coincided with the beginning of cellular swelling (Figure 1j and Supplementary Movie 1), suggesting the possibility that osmotically triggered water movement and succeeding cellular swelling, caused by increased ion influx, may drive the release of nucleosomes and DAMPs. These were tested using blockers of various ion channels in the plasma membrane. Very interestingly, DNA release was obstructed nearly completely by the Cl^−^ channel inhibitors, 4,4′-Diisothiocyanatostilbene-2,2′-disulfonic acid (DIDS) and 5-nitro-2-(3-phenylpropylamino) benzoic acid (NPPB), partially inhibited by the Na^+^ channel inhibitor, amiloride, and further prohibition of DNA release was observed by their combined use (Figure 6a), in accordance with decreased release of histones and DAMPs, whereas the inhibitors did not significantly affect cellular viability and death (Figures 6b–d). The effects were affirmed also in the condition of deprivation of amino acids (Supplementary Figures 6a–c). Requirement of Cl^−^ current in release of DNA was additionally validated by the cells in either Na^+^-free or Cl^−^-free medium, and subsequently DNA release was completely inhibited in Cl^−^-free medium (Figure 6e and Supplementary Figure 6d). Co-treatment of DIDS with staurosporine produced massive nuclear fragmentation (Figure 6f), increased TUNEL-positive population (Figure 6g) and inter-nucleosomal DNA fragmentation (Figure 6h). Therefore, hallmarks of apoptosis became apparent by inhibition of Cl^−^ channel. In fact, intracellular Cl^−^ concentration gradually increased with peak value at 10 h (Figure 6i) and Cl^−^ current also increased with a peak value 4 h after treatment of staurosporine (Figure 6j). Furthermore, DIDS partially suppressed Cl^−^ current, although amiloride produced little change (Figures 6k and l). However, the combined treatment of DIDS and amiloride completely blocked Cl^−^ current in staurosporine-treated cells (Figure 6m), indicating that the voltage gradient induced by Cl^−^ influx may have been neutralized by following Na^+^ influx, resulting in osmotically triggered movement of water and swelling. In addition, co-treatment of zVAD-fmk with staurosporine also inhibited Cl^−^ current (Figure 6n), indicating the probability that caspase activation may be associated with activation of Cl^−^ channels.

### Calpain activity is a prerequisite for nucleosome and DAMPs release

**Figure 7:**
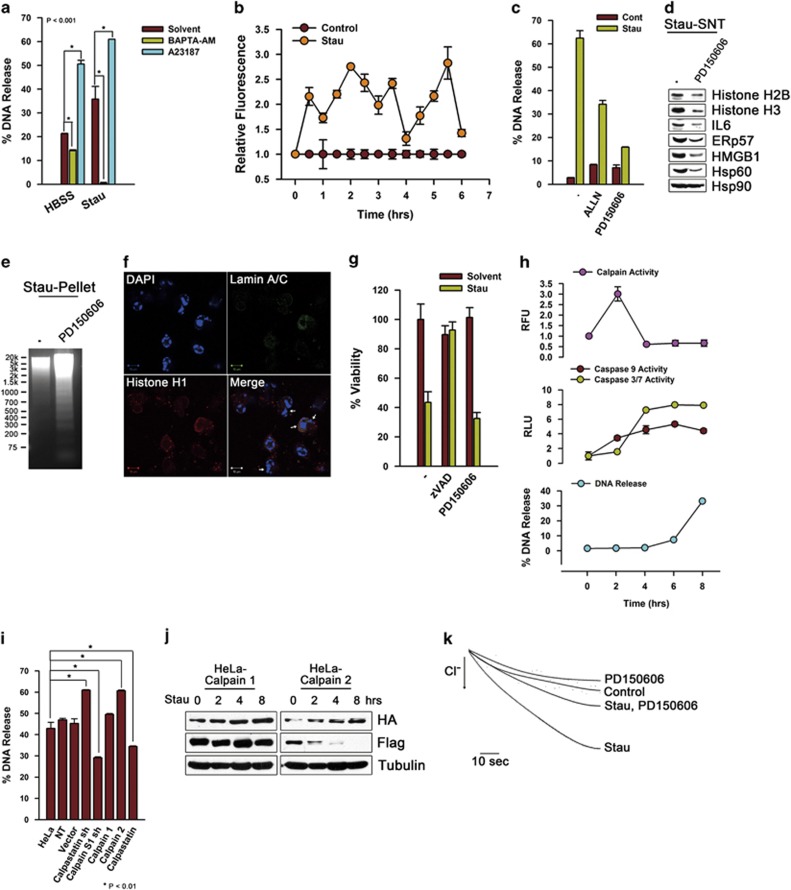
Calpain activity is a prerequisite for the release of nucleosomes and DAMPs in dying cells. HeLa cells were incubated in amino acid-depleted medium (*HBSS*) or staurosporine-containing medium (*Stau*) (1 *μ*g/ml) for 24 or 8 h, respectively, in the presence of solvent control, BAPTA-AM (50 *μ*M) or A23187 (1 *μ*g/ml). Released DNA was quantified with PicoGreen (**a**). Amount of intracellular free Ca^2+^ was measured in cells treated with solvent control or staurosporine at the indicated time periods by eFluor 514 (**b**). Cells were treated with staurosporine for 8 h in the presence of control solvent, ALLN (20 *μ*M), or PD150606 (20 *μ*M), and released DNA or protein was detected by PicoGreen staining (**c**) or western blot for histones and DAMPs (**d**), respectively. Genomic DNA was separated in agarose gel electrophoresis from staurosporine-treated cells with or without PD150606 (**e**). The cells treated with staurosporine and PD150606 for 8 h, were stained for lamin A/C and histone H1, and examined by confocal microscopy; the arrows indicate nuclear fragmentations (**f**). Cells treated with control solvent, zVAD-fmk (20 *μ*M), or PD150606 with or without staurosporine for 8 h were measured for viabilities with Calcein assay (**g**). Cells treated with staurosporine were measured for calpain activity, caspases 3 and 7 activity, or released DNAs at the indicated time periods (**h**). HeLa cells transfected with non-targeting shRNA (*NT*), calpastatin shRNA, calpain S1 shRNA, or overexpressing calpain 1, calpain 2, or calpastatin were incubated with staurosporine for 8 h and the released DNAs were measured by PicoGreen staining (**i**). Cells expressing calpain 1 or calpain 2 tagged with Flag at the N-terminus and HA at the C-terminus were treated with staurosporine and western-blotted with anti-Flag antibody or anti-HA antibody (**j**). HeLa cells expressing halide-sensitive YFP were treated with staurosporine and/or PD150606 for 4 h, and their chloride ion currents were measured by fluorometry by detecting quenching of YFP fluorescence (**k**). Data performed in triplicate are presented as mean±S.D. (**a**–**c** and **g**–**i**)

To evaluate the roles of intracellular free Ca^2+^, the effects of Ca^2+^ chelator, BAPTA-AM and the Ca^2+^ ionophore, A23187 were examined. BAPTA-AM reduced DNA release, whereas A23187 considerably increased DNA release in amino-acid-deprived or staurosporine-treated cells (Figure 7a). Furthermore, intracellular free Ca^2+^ was increased after staurosporine treatment (Figure 7b), implicating the presence of Ca^2+^-mediated effectors in nucleosomal release. Thus, we estimated the role of Ca^2+^-activated cysteine proteases, calpains because of their association with apoptotic and necrotic pathways.^11,12^ Calpain inhibitors ALLN or PD150606 partially blocked the release of nucleosomes and DAMPs except for Hsp90 (Figures 7c and d). Genomic DNA showed inter-nucleosomal fragmentation (Figure 7e), and nuclear condensation and fragmentation were produced upon co-treatment with PD150606 (Figure 7f) indicating that inhibition of calpain can convert necrotic cell death into apoptotic one. In contrast, there was no change in cellular viability (Figure 7g). Calpain activities were increased at 2 h, when DNA release and activities of caspases 3, 7 and 9 did not reach maximal levels, and decreased to the basal level by 4 h after staurosporine treatment (Figure 7h). Knock-down of calpastatin or overexpression of calpain 2 significantly increased DNA release, but knock-down of calpain S1 or overexpression of calpastatin considerably decreased DNA release (Figure 7i and Supplementary Figures 7a and b). In addition, calpain 2 began to show limited N-terminal degradation at 2 h after staurosporine treatment during the periods of increased calpain activities, whereas calpain 1 did not (Figure 7j), indicative of selective calpain II activation and its association with nucleosomal and DAMPs release. Regardless of previous reports revealing calpastatin degradation by caspases and subsequent calpain activation,^13, 14, 15^ calpastatin cleavage by caspases was not implicated in calpain activation, because DNA release from calpastatin mutant cells of caspases cleavage sites was not considerably different from that of calpastatin-overexpressing cells (Supplementary Figures 7c and 8a) and cleavage of calpastatin, although blocked by co-treatment of zVAD-fmk, only appeared in the beginning at 4 h at a time of no increased calpain activity (Supplementary Figure 8b). On the other hand, Cl^−^ currents were significantly inhibited by calpain inhibitors, indicating that one of possible roles of calpain could be activation of Cl^−^ channels (Figure 7k).

## Discussion

Recent advances in biology have differentiated regulated necrosis from the programmed cell death termed apoptosis.^16, 17, 18, 19^ Our data confirm that cell death involving the release of nucleosomes and DAMPs follows morphological necrosis (Figure 1), but is a caspase-dependent process (Figure 4). Therefore, the process should be classified as a kind of caspase-dependent regulated necrosis most importantly involving the conversion into apoptotic phenotypes by inhibition of the calpain pathway or Cl^−^ channel (Figures 6 and 7).

Necroptosis is a best understood caspase-independent regulatory necrosis, initiated by TNF*α–*TNFR1, FasL-Fas or Toll-like receptor pathway when apoptosis is blocked, requiring involvement of receptor interaction protein kinase 1 and 3 (RIPK1 and RIPK3).^20, 21, 22^ Execution phase of necroptosis has been largely unknown, however, implication of several elements including ROS, reactive nitrogen species, inhibition of mitochondrial adenine nucleotide translocase and phospholipase A2-lipoxigenase have been recently elucidated.^22,23^ Necroptosis and caspase-dependent regulated necrosis are presumed to be similar in morphological aspects, although both are different from each other in the requirements of caspases, RIPK1 or ROS (Figure 4 and Supplementary Figure 3). In addition, nucleosomal release in necroptosis is not reported, yet.

Apoptosis does not induce inflammation because its major regulators and processes have focused on eliminating pro-inflammatory materials.^24,25^ Contrastingly, the cell death shown here is completely at odds with this notion because caspases activation was indispensible for releasing the inflammatory materials. Although caspases function in initiating death processes and seem to be required for opening Cl^−^ channels, its precise molecular mechanisms remain unclear (Figures 4 and 6n).

DNA and nuclear DAMPs are reportedly known to be released in different types of cell death: late apoptosis or secondary necrosis, necrosis, or NETosis.^26^ Secondary necrosis is a necrotic change of terminal phase apoptotic cells when late apoptotic products are not adequately eliminated by nearby scavengers,^27,28^ from which extracellular nucleosome and DAMPs can be generated as membrane-bound vesicles after formation of apoptotic bodies,^29^ whereas DAMPs and nucleosomes are directly released in caspase-dependent necrosis as DAMPs bound poly-nucleosomes during relatively early phase when effector caspases are fully activated and mitochondrial membrane potential does not completely decline without apoptotic body formation and DNA fragmentation (Figure 1 and Supplementary Figure 1).

Major divergence of primary necrosis produced by H_2_O_2_ treatment or heating from caspase-dependent necrosis was a release of completely degraded DNA and detached DAMPs (Figures 3b and f). Moreover, some of DAMPs such as IL6 and ERp57 were not released in the primary necrosis (Figure 3c). Conclusively, both types of cell deaths are ultimately different from each other.

NETosis, neutrophil-specific form of generalized term ‘ETosis' is a kind of regulated necrosis, releasing neutrophil extracellular trap (NET) composed of chromatin, and cytoplasmic and granular products,^30^ and coordinated by Nox-generated ROS, PAD4-mediated chromatin decondensation and autophagy.^10,31^ In spite of its morphological similarities just as rapid, explosive dislodge of chromatin and attached components, the regulatory factors of NETosis were not connected to release of nucleosome and DAMPs in the caspase-dependent necrosis (Supplementary Figure 2).

Nucleosomal release is associated with various cytotoxic stimuli (Figure 2a) well-known to promote apoptosis by different mechanisms but absolutely depending on activities of caspases 3 and 9 (Figure 4 and Supplementary Figure 5). But nucleosomes were not always equally released in all the cells. For examples, HeLa and A549 showed relatively very high amount of DNA release compared with other cells, on the contrary, BEAS-2B, Huh7 and U937 showed little release of DNA (Figure 2b), suggesting the probability that cell death processes accompanying caspase activation can be subdivided into necrotic (caspase-dependent regulated necrosis) or apoptotic (apoptosis) at the execution phase in cell type-specific manners.

One candidate modulator determining the cell fates seems plausible to be calpain systems as its prohibition converted necrotic status to be completely apoptotic (Figures 7e and f) notwithstanding no acquaintance with its exact molecular mechanism. Calpains have been known to have an important role in caspase-dependent demises.^32, 33, 34, 35, 36, 37, 38, 39, 40, 41^ According to the data in Figure 7, calpain II was instantaneously activated early and its inhibition strikingly reduced nucleosomal release with no influence on cellular viability, indicating that instant early calpain activation is not crucial for cell death but needed for the imperative process implicated in nucleosomal and DAMPs release, possibly by regulating Cl^−^ channel activity. Consistent with this, it has been previously reported that calpains contribute to necrotic deaths through increase of plasma membrane permeability to ions, progressive disruption of cytoskeleton and plasma membrane proteins, and mitochondrial dysfunction.^42,43^

One of hallmarks of apoptosis is oligo-nucleosomal fragmentation orchestrated by DFF40/CAD. Nevertheless, this has been hardly detected in some types of cells despite the presence of effector caspase activation.^44, 45, 46, 47^ Moreover, it may not be an essential factor for apoptosis.^48, 49, 50, 51^ In this regard, our results reveal the probable role of CAD to execute its extracellular function as a bound form to released chromatin, leading to digestion of chromatin, although overexpression of CAD reduces nucleosomal release (Figures 5g–i).

Apoptotic volume decrease (AVD) concurrent with cell shrinkage is induced by activation of K^+^ and Cl^−^ channels at early apoptosis before caspase activation,^52^ whereas necrotic volume increase is caused by activation of Na^+^ channels.^53^ Dying HeLa cells initially showed classical AVD but promptly began to release their DNA with cellular swelling (Figure 1j and Supplementary Movie 1), accompanying the increase of intracellular free Cl^−^ concentration and Cl^−^ influx (Figures 6i and j). Thus, we postulate that osmotically triggered water flow with Cl^−^ influx is a major driving force for the release of nucleosomes and DAMPs, although, hitherto, the specific Cl^−^ channels are not yet certain. Cl^−^ channels have been demonstrated to function mainly in cellular volume regulation and fluid secretion. Recent findings also have shown their close connection with various human diseases.^54,55^

**Figure 8:**
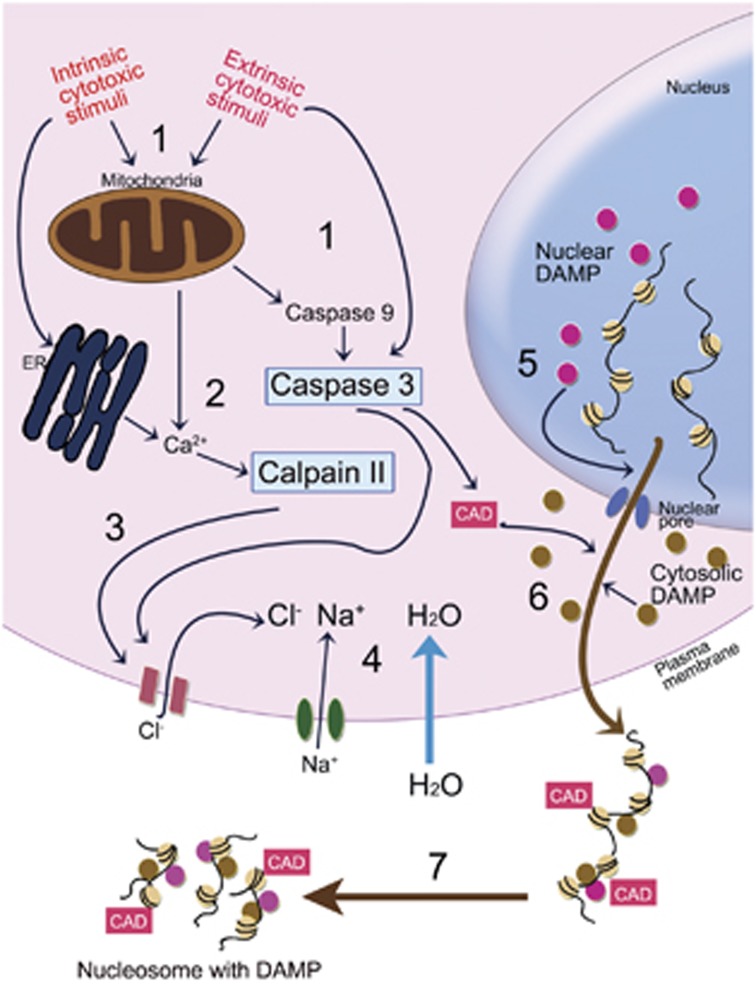
Schematic diagram showing the suggested molecular mechanisms of caspase-dependent regulated necrosis. Caspases 9 and 3 are sequentially activated by various cytotoxic stimuli (1). At the same time, focally released Ca^2+^ from either ER or mitochondria in the cellular stress activates calpain II (2), which opens Cl^−^ ion channels of plasma membrane cooperating together with caspase 3 by unknown mechanisms (3). Cl^−^ influx and secondary Na^+^ influx, mediated by voltage gradient produced by Cl^−^ influx, induce osmotically driven water movement into cytosol and subsequent cellular swelling (4). Hydrostatic pressure, generated by intracellular water increment, squeezes out nuclear chromatin through nuclear pore damaged by caspases to be released extracellularly by way of cytosol. During that time, nuclear and cytosolic DAMPs and discharged CAD from ICAD by caspase 3 are attached to the released chromatin (5, 6). Finally, the extracellular chromatin and its attached DAMPs are partially digested into poly- or oligo-nucleosomes with DAMPs by the CAD (7)

In conclusion, we propose a novel entity of cellular demise, caspase-dependent regulated necrosis. It can be defined as follows (Figure 8): characterized by massive release of poly- and oligo-nucleosomes and their attached DAMPs and displaying necrotic morphology with no fragmentation of nuclei and DNA; subdivided from apoptosis at the step of caspases 9 and 3 activation, hence, the cell death can be blocked by zVAD-fmk; instant activation of calpains is needed for release of nucleosomes and DAMPs, subsequently, inhibition of calpain convert the necrotic cellular demise to the apoptotic one; osmotically triggered water movement following Cl^−^ influx and secondary Na^+^ influx is a critical factor, possibly through providing the driving force for nucleosomal and DAMPs release.

## Materials and Methods

### Cells, antibodies and other reagents

HeLa cell line (human cervical cancer) was cultured in minimal essential media (MEM) supplemented with 10% fetal bovine serum (FBS), 2 mM L-glutamine, 100 U/ml penicillin and 100 *μ*g/ml streptomycin. HCT116 and HCT116 Bax (−/−) (human colon cancer), U87MG (human malignant glioma), U251MG (human neuronal glioblastoma), Hep3B, HepG2, and Huh7 (human hepatoma), DU145 and PC3 (human prostatic cancer), MDA-MB231 and MCF7 (human mammary cancer), HEK 293T (human kidney epithelium) cell lines were cultured in DMEM supplemented with 10% FBS, 2 mM L-glutamine, 100 U/ml penicillin and 100 *μ*g/ml streptomycin. U937 (human histiocytic lymphoma) was cultured in RPMI1640 supplemented with 10% FBS, 2 mM L-glutamine, 100 U/ml penicillin and 100 *μ*g/ml streptomycin. SKOV3 and SKOV3ip (human ovarian adenocarcinoma) was cultured in RPMI1640 supplemented with 20% FBS, 2 mM L-glutamine, 100 U/ml penicillin and 100 *μ*g/ml streptomycin. Staurosporine and etoposide (VP16) were purchased from Cell Signaling Technology (Danvers, MA, USA). Cycloheximide was purchased from Calbiochem (Darmstadt, Germany). Human TNF*α* was purchased from R&D Systems (Minneapolis, MN, USA). Cl-amidine was purchased from Cayman Chemical (Ann Arbor, MI, USA). zVAD-fmk, zDEVD-fmk, Necrostatin-1, BAPTA-AM, diphenyliodonium, PD150606, ALLN and A23187 were purchased from Santa Cruz Biotechnology (Santa Cruz, CA, USA). DNase I, *N*-acetyl cysteine (NAC), ascorbic acid, 3MA and AG14699 were from Sigma-Aldrich (Yongin, Korea). An inhibitor of epithelial Na^+^ channel, amiloride hydrochloride hydrate; Cl^−^ channel inhibitors, DIDS and NPPB; an inhibitor of Na^+^/Cl^−^ cotransporter, hydrochlorothiazide (HCT); an inhibitor of Na^+^ K^+^ ATPase, ouabain; an inhibitor of non-selective cation channel, flufenamide; an inhibitor of stretch-activated ion channel, Gad (III) chloride hexahydrate; an inhibitor of K^+^ channel, tetraethyl ammonium chloride (TEA); and an inhibitor of Na^+^/K^+^ cotransporter, bumetanide were purchased from Sigma-Aldrich. Na^+^-free media was made by substituting NaCl with *N*-methyl-D-glucamine (Sigma-Aldrich) from MEM media or Hank's balanced salt solution (HBSS). Cl^−^ medium was made by substituting KCl, KH_2_PO_4_ and CaCl_2_ with sodium gluconate, potassium gluconate and calcium acetate hydrate (Sigma-Aldrich). Anti-histones (H1, H2A, H2B, H3 and H4), anti-ERp57, anti-IL6, anti-lamin A/C and anti-hemagglutinin tag (HA) antibodies were purchased from Santa Cruz Biotechnology. Anti-Hsp60, anti-Hsp90, anti-PARP, anti-caspase 3, anti-caspase 6, anti-caspase 7, anti-caspase 9, anti-ICAD, anti-calpastatin, anti-calpain 1 and anti-calpain 2 antibodies were purchased from Cell Signaling Technology; anti-HMGB1 antibody from Abcam (Cambridge, MA, USA); and anti-*α*-tubulin antibody from Calbiochem. Anti-CAD antibody was purchased from Novus Biologicals (Littleton, CO, USA). Anti-calpain S1 antibody was purchased from Thermo Scientific (Seoul, Korea). Anti-Flag tag and anti-HA tag antibodies were purchased from Sigma-Aldrich.

### Expression constructs and lentiviral transfections

Lentiviral constructs expressing shRNAs for caspases 1, 3, 6, 7, 9, CAD, EndoG, calpastatin, calpain S1, PAD2, PAD4, Atg5, Vps34 and Beclin1 were purchased from Sigma-Aldrich. cDNAs of caspase 3, CAD, EndoG, calpain 1 and calpain 2 were purchased from OriGene (Rockville, MD, USA), calpastatin cDNA was from Shi-Yong Sun (Emory University School of Medicine), being subcloned into pCDH-EF2-MCS-T2A-Puro, a lentiviral vector for cDNA expression (System Biosciences, Mountain View, CA, USA). Mutation of calpastatin at cleavage sites of caspases was accomplished using a QuickChange site-directed mutagenesis kit (Stratagene, Santa Clara, CA, USA). All the lentiviral vectors were transfected to 293TN cells (System Biosciences) with Lipofectamine 2000 transfection reagent (Invitrogen, Seoul, Korea). Particles were collected 2 days after the transfection of lentiviral plasmids, and infected into the cells. Lentivirus-infected cells were puromycin-selected for 1 week.

### Polymerase chain reaction (PCR)

The released DNAs and cellular genomic DNAs were PCR amplified using primers for genomic sequences; *GAPDH* (5′-CCCCTTCATTGACCTCAACTAC-3′ and 5′-GAGTCCTTCCACGATACCAAAG-3′), *FAS* (5′-TCACCACTATTGCTGGAGTCAT-3′ and 5′-TAAACATCCTTGGAGGCAGAAT-3′), and the released DNAs and mitochondrial DNAs were PCR-amplified using primers for mitochondrial genes; ATP synthase subunit 6 (*ATP6*) (5′-ATACACAACACTAAAGGACGAACCT-3′ and 5′-GAGGCTTACTAGAAGTGTGAAAACG-3′), cytochrome oxidase c subunit 1 *(CO1)* (5′-GGAGTCCTAGGCACAGCTCTAA-3′ and 5′-GGAGGGTAGACTGTTCAACCTG-3′) for determining the presence of genomic and mitochondrial gene sequences in the released DNAs of dying cancer cells.^56^

### Preparation of cell lysates and western blots

The conditioned medium from HBSS-incubated or staurosporine-treated cells were 100-fold concentrated with Amicon Ultra Centrifugal Filters (Millipore, Darmstadt, Germany; 3000 Da MW cut-off). For preparing total cell lysates, cells were lysed in high salt lysis buffer (50 mM HEPES (pH 7.5), 250 mM NaCl, 1% Triton X-100, 1 mM EDTA, 1 mM dithiothreitol, 1 mM Na_3_VO_4_, 1 mM NaF, 1 *μ*g/ml pepstatin A, 10 *μ*g/ml AEBSF, 2 *μ*g/ml aprotinin and 1 *μ*g/ml leupeptin), incubated on ice for 20 min and centrifuged for 20 min to remove cell debris. The concentrated conditioned media or total cell lysate was subjected to sodium dodecyl sulfate-polyacrylamide electrophoresis. The proteins were then electro-transferred to PVDF membranes and incubated overnight with antibodies at 4 °C. Subsequently, the membranes were incubated with peroxidase-conjugated secondary antibodies (Pierce, Rockford, IL, USA) for 1 h at room temperature, and the signal was detected using an enhanced chemiluminescence (ECL) detection kit (Amersham Biosciences, Seongnam, Korea).

### Quantification of released DNAs

Cells were cultured in six-well plates for >24 h to 80% confluency and treated with various cytotoxic reagents for the indicated time periods. Released DNA from dying cells were digested with 500 mU/ml MNase (Thermo Scientific) for 5 min. Nuclease activity was stopped with 5 mM EDTA and the culture supernatants were collected and stored at −20 °C until quantification. Total genomic DNA from HeLa cells was extracted with 500 *μ*l DNazol (Molecular Research Center, Cincinnati, OH, USA). Total DNA and released DNA were quantified using a PicoGreen dsDNA assay kit (Life Technologies, Seoul, Korea) according to the manufacturer's instructions. Data are presented as % DNA release calculated as (released DNA/total genomic DNA) × 100.^57^

### Live cell fluorescence imaging

Cells on Lab-Tek two-well glass chamber slide (NUNC, Rockford, IL, USA) were incubated in the presence of membrane-impermeable DNA dye, SYTOX Green (50 nM; Life Technologies), and membrane-permeable DNA dye DRAQ5 (5 *μ*M; Cell Signaling Technology). The images were acquired every 5 min for 24 h via a 100 × objective on a Deltavision RT Deconcolution microscope (Applied Precision, Issaquah, WA, USA) using a Photometrics Cool SNAPHQ^2^ camera (Roper-Princeton Instruments, Trenton, NJ, USA) controlled by SoftWoRxTM Imaging workstation (Applied Precision).

### Confocal microscopy

Cells grown on Lab-Tek four-well glass chamber slides (NUNC) were incubated in HBSS or medium containing appropriate reagents for the indicated times. Cells were fixed with 4% paraformaldehyde and permeabilized with 0.2% Triton X-100 for 5 min. They were washed with PBS and incubated with primary antibodies and subsequently with secondary antibody conjugates (Alexa Fluor 594 donkey anti-mouse IgG and/or Alexa Fluor 488 donkey anti-rabbit IgG; Invitrogen). Images were collected using a laser scanning confocal microscope LSM710 (Carl Zeiss, Oberkochen, Germany) equipped with argon (488 nm) and krypton (568 nm) lasers, using an x40 water immersion objective. Images were processed with ZEN 2009 light edition (Carl Zeiss).

### Transmission electron microscopy

The cells were pelleted and washed twice with PBS. Fixation was performed with phosphate buffer pH 7.4 containing 2.5% glutaraldehyde for 30 min at 4 °C. The pellets were rinsed twice with cold PBS, post-fixed in buffered OsO_4_, dehydrated in graded acetone and embedded in Durcupan ACM resin (Fluka, Yongin, Korea). Ultrathin sections were obtained, mounted in copper grids and counterstained with uranyl acetate and lead citrate. The specimens were observed with a Hitachi H-7600 TEM (Schaumburg, IL, USA) at 80 kV.

### Assays for cellular viability and cell death, TUNEL staining, Annexin V staining and JC-1 staining

Cellular viability was quantified with Calcein-AM (Invitrogen) as specified by the manufacturer. The cells were aliquoted into 96-black well plates, incubated with Calcein-AM (2.5 *μ*M) for 15 min and fluorescent intensities were measured at excitation and emission wavelength of 490 nm and 520 nm, respectively. Data were the mean±S.D. of four independent measurements, and are presented as % viability ((fluorescence intensity of treated cells/fluorescent intensity of non-treated cells) × 100). Assays for determining cell death were done using cell-impermeable DNA dyes, PI (Calbiochem) or SYTOX Green (Life Technologies). Cells in triplicate were washed, stained briefly with PI (500 nM) or SYTOX Green (30 nM), and fluorescence intensities were analyzed by flow cytometry, and are presented as % cell death ((dead cellular counts/total cellular counts) × 100). Intracellular DNA fragmentation was detected using APO-BrdU TUNEL assay kit (Invitrogen) as specified by the manufacturer. Suspensions of dying cells were sequentially fixed by adding 1% paraformaldehyde in PBS for 15 min and 70% ethanol for 30 min in ice, washed twice, and DNA-labeled with TdT enzyme and BrdUTP for 60 min at 37 °C. The cells were stained with Alexa Fluor 488-conjugated anti-BrdU antibody for 30 min at room temperature and with PI/RNase A for 30 min at room temperature, and finally analyzed by flow cytometry. Mitochondrial membrane potential was measured by staining of a membrane-permeable JC-1 dye (MACS Miltenyi Biotech, Bergisch Gladbach, Germany) and analysis by flow cytometry. Annexin V staining was performed for detecting the exposure of phosphatidylserine to outer leaflet of plasma membrane that is a marker of early apoptosis using a Biotin Annexin V staining kit (BD Biosciences, San Jose, CA, USA) according to the manufacturer's protocol. Annexin V-positive population was defined when Annexin V staining was positive and PI staining was negative in the population by flow cytometry.

### ChIP assay

The assay was performed using EZ-Magna ChIP A/G assay kit (Millipore) according to the manufacturer's instructions with minor modifications. Briefly, HeLa cells were treated with 1 *μ*g/ml staurosporine for 10 h, with 32 mM H_2_O_2_ for 4 h, or heated at 65 °C for 1 h and incubated for 3 h. The conditioned media were fixed with 1% formaldehyde for 10 min, quenched with 1X glycine for 5 min and concentrated. The concentrated media were incubated overnight with goat polyclonal anti-histone H1 antibody (Santa Cruz Biotechnology), goat polyclonal anti-HMGB1 antibody (Santa Cruz Biotechnology) or normal goat serum. The antibody–protein–DNA complexes were captured by incubation with EZ-Magna ChIP A/G. Genomic DNA segments from complexes were eluted, digested with proteinase K digestion and purified using spin columns. Aliquots of the DNA were used as a template for PCR amplification using 35 cycles of 55 °C annealing temperature. The *GAPDH* primers for PCR amplification were 5′-CCCCTTCATTGACCTCAACTAC-3′ and 5′-GAGTCCTTCCACGATACCAAAG-3′.

### Caspase activity assay

Activities of caspases 3/7 and 9 were measured by Caspase-Glo 3/7 Assay and Caspase-Glo 9 Assay (Promega, Seoul, Korea) according to manufacturer's instructions.

### Calpain assay

Calpain activity was measured using Calpain Activity Fluorometric Assay Kit (Biovision, San Francisco, CA, USA) according to the manufacturer's instructions.

### DNA laddering analysis

The cells were pelleted, washed twice and DNA extracted with DNazol (Molecular Research Center). DNA was precipitated with two volumes of ethanol and 0.1 volumes of 3 M sodium acetate, pH 5.2. DNA released from dying cells was concentrated, purified by phenol–chloroform extraction and precipitated with two volume of ethanol and 0.1 volumes of 3 M sodium acetate, pH 5.2. The precipitated DNA was resuspended in TE buffer pH 8.0 containing DNase-free RNase, and analyzed using 1.5% agarose gel in 1 × TBE buffer.

### Measurements of intracellular chloride ion concentration

Fluorescence-based microplate assay applying intracellular quenching of *N*-(ethoxycarbonylmethyl)-6-methoxyquinolinium bromide (MQAE) fluorescence was used for quantifying intracellular Cl^−^ concentrations as described previously.^58^ HeLa cells loaded with 5 mM MQAE (Life Technologies) overnight were incubated with or without staurosporine (1 *μ*g/ml) for the indicated times. One set of the cells was washed three times with Cl^−^ containing buffer (HBSS) and MQAE fluorescence was measured at excitation wavelength of 360 nm and an emission wavelength of 460 nm (F_t_), and the other set of the cells were washed three times with Cl^−^-free HBSS, suspended with Cl^−^-free HBSS containing 10 *μ*M tributyltin chloride (Sigma-Aldrich) and 5 *μ*M nigericin sodium salt (Sigma-Aldrich). MQAE fluorescence was measured (F_0_). F_0_/F_t_ values were used as representative values for intracellular Cl^−^ concentrations according to Stern–Volmer equation (F_0_/F_t_=1+K_Cl_ [Cl], where [Cl] is the intracellular Cl^−^ concentrations and K_Cl_ is the Stern–Volmer constant).

### Measurements of chloride ion currents using halide-sensitive YFP

Cellular Cl^−^ currents were measured using Premo Halide Sensor (Invitrogen) as specified by the manufacturer. Briefly, HeLa cells infected with baculovirus encoding halide-sensitive yellow fluorescent protein (YFP) were incubated with various reagents for indicated times, mixed with an equal volume of 2X Premo halide stimulus buffer containing 150 mM NaI and the fluorescence intensities were monitored every 2 s   for 60 s at an excitation wavelength of 480 nm and an emission wavelength of 560 nm to record YFP quenching evoked by inward currents of iodide ion.

### Measurements of intracellular free calcium ion

Intracellular free Ca^2+^ was measured by Calcium Sensor Dye eFluor 514 (eBiosciences, San Diego, CA, USA). Briefly, cells were incubated for 30 min at 37 °C in medium containing 5 *μ*M eFluor 514 and washed twice. Fluorescence was measured at an excitation wavelength of 490 nm and an emission wavelength of 514 nm with a FLUOstar Optima Microplate Fluorometer (BMG Labtech, Cary, NC, USA). Data were presented as relative fluorescence; MFI of treated cells/MFI of non-treated cells.

### Real-time PCR

Total RNA was isolated using an RNeasy kit (Qiagen, Seoul, Korea). PrimeScript RT reagent Kit (TaKaRa, Seoul, Korea) was used to reverse transcribe mRNA into cDNA. PCR was then performed on an ABI PRISM 7000 machine (Applied Biosystems, Carlsbad, CA, USA) using SYBR Premix Ex Taq II (TaKaRa). The sequences of primers for *PAD2* and *PAD4* were: *PAD2* (5′-GTGACAACCCTCGGTGTGGA-3′ and 5′-ACATCAAGGTGGAAGCAGGAACTTA-3′), *PAD4* (5′-GAGGCTGTGGTGTTCCAAGA-3′ and 5′-TCAGCTTGCACTTGGCTTTC-3′). Analysis of each sample was performed more than twice for each experiment, and data in the figures are reported as relative quantification: average values of 2^−ΔΔCT^±S.D.

### Statistical analysis

All the values are presented as mean±S.E. A paired Student's *t-*test was used to identify significant differences in comparisons. A level of *P*<0.05 was considered statistically significant.

## Abbreviations

3MA: 3-methyl adenine
DAMP: damage-associated molecular pattern
DFF40/CAD: caspase-activated DNase
DIDS: 4,4′-Diisothiocyanatostilbene-2,2′-disulfonic acid
EndoG: endonuclease G
HMGB1: high mobility group protein B1
Hsp90: heat shock protein 90
ICAD: inhibitor of caspase-activated DNase
IL6: interleukin 6
MEM: minimal essential media
MNase: micrococcal nuclease
NAC: *N*-acetyl cysteine
NPPB: 2-(3-phenylpropylamino) benzoic acid
PAD: peptidylarginine deaminase
PARP-1: poly ADP ribose polymerase-1
shRNA: short hairpin RNA

## References

[bib1] 1Galluzzi L, Vitale I, Abrams JM, Alnemri ES, Baehrecke EH, Blagosklonny MV et al. Molecular definitions of cell death subroutines: recommendations of the Nomenclature Committee on Cell Death 2012. Cell Death Differ 2011; 19: 107–120. 21760595 10.1038/cdd.2011.96 PMC3252826

[bib2] 2Taylor RC, Cullen SP, Martin SJ. Apoptosis: controlled demolition at the cellular level. Nat Rev Mol Cell Biol 2008; 9: 231–241. 18073771 10.1038/nrm2312

[bib3] 3Kitanaka C, Kuchino Y. Caspase-independent programmed cell death with necrotic morphology. Cell Death Differ 1999; 6: 508–515. 10381653 10.1038/sj.cdd.4400526

[bib4] 4Martin SJ, Henry CM, Cullen SP. A perspective on mammalian caspases as positive and negative regulators of inflammation. Mol Cell 2012; 46: 387–397. 22633487 10.1016/j.molcel.2012.04.026

[bib5] 5Proskuryakov SY, Konoplyannikov AG, Gabai VL. Necrosis: a specific form of programmed cell death? Exp Cell Res 2003; 283: 1–16. 12565815 10.1016/s0014-4827(02)00027-7

[bib6] 6Storr SJ, Carragher NO, Frame MC, Parr T, Martin SG. The calpain system and cancer. Nat Rev Cancer 2011; 11: 364–374. 21508973 10.1038/nrc3050

[bib7] 7Fiers W, Beyaert R, Declercq W, Vandenabeele P. More than one way to die: apoptosis, necrosis and reactive oxygen damage. Oncogene 1999; 18: 7719–7730. 10618712 10.1038/sj.onc.1203249

[bib8] 8Yoon S, Woo SU, Kang JH, Kim K, Kwon MH, Park S et al. STAT3 transcriptional factor activated by reactive oxygen species induces IL6 in starvation-induced autophagy of cancer cells. Autophagy 2010; 6: 1125–1138. 20930550 10.4161/auto.6.8.13547

[bib9] 9Yoon S, Woo SU, Kang JH, Kim K, Shin HJ, Gwak HS et al. NF-kB and STAT3 cooperatively induce IL6 in starved cancer cells. Oncogene 2012; 31: 3467–3481. 22105366 10.1038/onc.2011.517

[bib10] 10Remijsen Q, Kuijpers TW, Wirawan E, Lippens S, Vandenabeele P, Vanden Berghe T. Dying for a cause: NETosis, mechanisms behind an antimicrobial cell death modality. Cell Death Differ 2011; 18: 581–588. 21293492 10.1038/cdd.2011.1 PMC3131909

[bib11] 11Vandenabeele P, Orrenius S, Zhivotovsky B. Serine proteases and calpains fulfill important supporting roles in the apoptotic tragedy of the cellular opera. Cell Death Differ 2005; 12: 1219–1224. 16094402 10.1038/sj.cdd.4401719

[bib12] 12Zong WX, Thompson CB. Necrotic death as a cell fate. Genes Dev 2006; 20: 1–15. 16391229 10.1101/gad.1376506

[bib13] 13Kato M, Nonaka T, Maki M, Kikuchi H, Imajoh-Ohmi S. Caspases cleave the amino-terminal calpain inhibitory unit of calpastatin during apoptosis in human Jurkat T cells. J Biochem 2000; 127: 297–305. 10731697 10.1093/oxfordjournals.jbchem.a022607

[bib14] 14Wang KK, Posmantur R, Nadimpalli R, Nath R, Mohan P, Nixon RA et al. Caspase-mediated fragmentation of calpain inhibitor protein calpastatin during apoptosis. Arch Biochem Biophys 1998; 356: 187–196. 9705209 10.1006/abbi.1998.0748

[bib15] 15Porn-Ares MI, Samali A, Orrenius S. Cleavage of the calpain inhibitor, calpastatin, during apoptosis. Cell Death Differ 1998; 5: 1028–1033. 9894609 10.1038/sj.cdd.4400424

[bib16] 16Galluzzi L, Kepp O, Krautwald S, Kroemer G, Linkermann A. Molecular mechanisms of regulated necrosis. Semin Cell Dev Biol 2014; 35: 24–32. 24582829 10.1016/j.semcdb.2014.02.006

[bib17] 17Kaczmarek A, Vandenabeele P, Krysko DV. Necroptosis: the release of damage-associated molecular patterns and its physiological relevance. Immunity 2013; 38: 209–223. 23438821 10.1016/j.immuni.2013.02.003

[bib18] 18Wang Y, Dawson VL, Dawson TM. Poly(ADP-ribose) signals to mitochondrial AIF: a key event in parthanatos. Exp Neurol 2009; 218: 193–202. 19332058 10.1016/j.expneurol.2009.03.020 PMC2752872

[bib19] 19Wang Y, Kim NS, Haince JF, Kang HC, David KK, Andrabi SA et al. Poly(ADP-ribose) (PAR) binding to apoptosis-inducing factor is critical for PAR polymerase-1-dependent cell death (parthanatos). Sci Signal 2011; 4: ra20. 21467298 10.1126/scisignal.2000902 PMC3086524

[bib20] 20Giampietri C, Starace D, Petrungaro S, Filippini A, Ziparo E. Necroptosis: molecular signalling and translational implications. Int J Cell Biol 2014; 2014: 490275. 24587805 10.1155/2014/490275 PMC3920604

[bib21] 21Linkermann A, Green DR. Necroptosis. N Engl J Med 2014; 370: 455–465. 24476434 10.1056/NEJMra1310050 PMC4035222

[bib22] 22Wu W, Liu P, Li J. Necroptosis: an emerging form of programmed cell death. Crit Rev Oncol Hematol 2012; 82: 249–258. 21962882 10.1016/j.critrevonc.2011.08.004

[bib23] 23Vandenabeele P, Galluzzi L, Vanden Berghe T, Kroemer G. Molecular mechanisms of necroptosis: an ordered cellular explosion. Nat Rev Mol Cell Biol 2010; 11: 700–714. 20823910 10.1038/nrm2970

[bib24] 24Kono H, Rock KL. How dying cells alert the immune system to danger. Nat Rev Immunol 2008; 8: 279–289. 18340345 10.1038/nri2215 PMC2763408

[bib25] 25Matzinger P. Tolerance, danger, and the extended family. Annu Rev Immunol 1994; 12: 991–1045. 8011301 10.1146/annurev.iy.12.040194.005015

[bib26] 26Pisetsky DS. The origin and properties of extracellular DNA: from PAMP to DAMP. Clin Immunol 2012; 144: 32–40. 22659033 10.1016/j.clim.2012.04.006 PMC3724456

[bib27] 27Silva MT. Secondary necrosis: the natural outcome of the complete apoptotic program. FEBS Lett 2010; 584: 4491–4499. 20974143 10.1016/j.febslet.2010.10.046

[bib28] 28Silva MT, do Vale A, dos Santos NM. Secondary necrosis in multicellular animals: an outcome of apoptosis with pathogenic implications. Apoptosis 2008; 13: 463–482. 18322800 10.1007/s10495-008-0187-8 PMC7102248

[bib29] 29Beyer C, Stearns NA, Giessl A, Distler JH, Schett G, Pisetsky DS. The extracellular release of DNA and HMGB1 from Jurkat T cells during *in vitro* necrotic cell death. Innate Immun 2012; 18: 727–737. 22344226 10.1177/1753425912437981 PMC3724467

[bib30] 30Brinkmann V, Reichard U, Goosmann C, Fauler B, Uhlemann Y, Weiss DS et al. Neutrophil extracellular traps kill bacteria. Science 2004; 303: 1532–1535. 15001782 10.1126/science.1092385

[bib31] 31Vanden Berghe T, Linkermann A, Jouan-Lanhouet S, Walczak H, Vandenabeele P. Regulated necrosis: the expanding network of non-apoptotic cell death pathways. Nat Rev Mol Cell Biol 2014; 15: 135–147. 24452471 10.1038/nrm3737

[bib32] 32Al-Bahlani S, Fraser M, Wong AY, Sayan BS, Bergeron R, Melino G et al. P73 regulates cisplatin-induced apoptosis in ovarian cancer cells via a calcium/calpain-dependent mechanism. Oncogene 2011; 30: 4219–4230. 21516125 10.1038/onc.2011.134 PMC3194400

[bib33] 33Del Bello B, Moretti D, Gamberucci A, Maellaro E. Cross-talk between calpain and caspase-3/-7 in cisplatin-induced apoptosis of melanoma cells: a major role of calpain inhibition in cell death protection and p53 status. Oncogene 2007; 26: 2717–2726. 17130844 10.1038/sj.onc.1210079

[bib34] 34Gil-Parrado S, Fernandez-Montalvan A, Assfalg-Machleidt I, Popp O, Bestvater F, Holloschi A et al. Ionomycin-activated calpain triggers apoptosis. A probable role for Bcl-2 family members. J Biol Chem 2002; 277: 27217–27226. 12000759 10.1074/jbc.M202945200

[bib35] 35Kim JS, Lee JH, Jeong WW, Choi DH, Cha HJ, Kim do H et al. Reactive oxygen species-dependent EndoG release mediates cisplatin-induced caspase-independent apoptosis in human head and neck squamous carcinoma cells. Int J Cancer 2008; 122: 672–680. 17955488 10.1002/ijc.23158

[bib36] 36Ben-Aharon I, Gafter-Gvili A, Paul M, Leibovici L, Stemmer SM. Interventions for alleviating cancer-related dyspnea: a systematic review. J Clin Oncol 2008; 26: 2396–2404. 18467732 10.1200/JCO.2007.15.5796

[bib37] 37Gao G, Dou QP. N-terminal cleavage of bax by calpain generates a potent proapoptotic 18-kDa fragment that promotes bcl-2-independent cytochrome C release and apoptotic cell death. J Cell Biochem 2000; 80: 53–72. 11029754 10.1002/1097-4644(20010101)80:1<53::aid-jcb60>3.0.co;2-e

[bib38] 38Jang YM, Kendaiah S, Drew B, Phillips T, Selman C, Julian D et al. Doxorubicin treatment *in vivo* activates caspase-12 mediated cardiac apoptosis in both male and female rats. FEBS Lett 2004; 577: 483–490. 15556633 10.1016/j.febslet.2004.10.053

[bib39] 39Liu L, Xing D, Chen WR. Micro-calpain regulates caspase-dependent and apoptosis inducing factor-mediated caspase-independent apoptotic pathways in cisplatin-induced apoptosis. Int J Cancer 2009; 125: 2757–2766. 19705411 10.1002/ijc.24626

[bib40] 40Liu L, Xing D, Chen WR, Chen T, Pei Y, Gao X. Calpain-mediated pathway dominates cisplatin-induced apoptosis in human lung adenocarcinoma cells as determined by real-time single cell analysis. Int J Cancer 2008; 122: 2210–2222. 18214855 10.1002/ijc.23378

[bib41] 41Mandic A, Viktorsson K, Strandberg L, Heiden T, Hansson J, Linder S et al. Calpain-mediated Bid cleavage and calpain-independent Bak modulation: two separate pathways in cisplatin-induced apoptosis. Mol Cell Biol 2002; 22: 3003–3013. 11940658 10.1128/MCB.22.9.3003-3013.2002 PMC133754

[bib42] 42Liu X, Van Vleet T, Schnellmann RG. The role of calpain in oncotic cell death. Annu Rev Pharmacol Toxicol 2004; 44: 349–370. 14744250 10.1146/annurev.pharmtox.44.101802.121804

[bib43] 43Waters SL, Sarang SS, Wang KK, Schnellmann RG. Calpains mediate calcium and chloride influx during the late phase of cell injury. J Pharmacol Exp Ther 1997; 283: 1177–1184. 9399991

[bib44] 44Boix J, Llecha N, Yuste VJ, Comella JX. Characterization of the cell death process induced by staurosporine in human neuroblastoma cell lines. Neuropharmacology 1997; 36: 811–821. 9225309 10.1016/s0028-3908(97)00030-0

[bib45] 45Iguchi K, Hirano K, Ishida R. Activation of caspase-3, proteolytic cleavage of DFF and no oligonucleosomal DNA fragmentation in apoptotic Molt-4 cells. J Biochem 2002; 131: 469–475. 11872177 10.1093/oxfordjournals.jbchem.a003123

[bib46] 46Sikora E, Bielak-Zmijewska A, Magalska A, Piwocka K, Mosieniak G, Kalinowska M et al. Curcumin induces caspase-3-dependent apoptotic pathway but inhibits DNA fragmentation factor 40/caspase-activated DNase endonuclease in human Jurkat cells. Mol Cancer Ther 2006; 5: 927–934. 16648563 10.1158/1535-7163.MCT-05-0360

[bib47] 47Yuste VJ, Bayascas JR, Llecha N, Sanchez-Lopez I, Boix J, Comella JX. The absence of oligonucleosomal DNA fragmentation during apoptosis of IMR-5 neuroblastoma cells: disappearance of the caspase-activated DNase. J Biol Chem 2001; 276: 22323–22331. 11294834 10.1074/jbc.M100072200

[bib48] 48Jacobsen MD, Weil M, Raff MC. Role of Ced-3/ICE-family proteases in staurosporine-induced programmed cell death. J Cell Biol 1996; 133: 1041–1051. 8655577 10.1083/jcb.133.5.1041 PMC2120856

[bib49] 49Jacobson MD, Burne JF, Raff MC. Programmed cell death and Bcl-2 protection in the absence of a nucleus. EMBO J 1994; 13: 1899–1910. 8168488 10.1002/j.1460-2075.1994.tb06459.x PMC395031

[bib50] 50Schulze-Osthoff K, Walczak H, Droge W, Krammer PH. Cell nucleus and DNA fragmentation are not required for apoptosis. J Cell Biol 1994; 127: 15–20. 7523418 10.1083/jcb.127.1.15 PMC2120176

[bib51] 51Iglesias-Guimarais V, Gil-Guinon E, Gabernet G, Garcia-Belinchon M, Sanchez-Osuna M, Casanelles E et al. Apoptotic DNA degradation into oligonucleosomal fragments, but not apoptotic nuclear morphology, relies on a cytosolic pool of DFF40/CAD endonuclease. J Biol Chem 2012; 287: 7766–7779. 22253444 10.1074/jbc.M111.290718 PMC3293563

[bib52] 52Bortner CD, Cidlowski JA. Apoptotic volume decrease and the incredible shrinking cell. Cell Death Differ 2002; 9: 1307–1310. 12478467 10.1038/sj.cdd.4401126

[bib53] 53Barros LF, Hermosilla T, Castro J. Necrotic volume increase and the early physiology of necrosis. Comp Biochem Physiol A Mol Integr Physiol 2001; 130: 401–409. 11913453 10.1016/s1095-6433(01)00438-x

[bib54] 54Duran C, Thompson CH, Xiao Q, Hartzell HC. Chloride channels: often enigmatic, rarely predictable. Annu Rev Physiol 2010; 72: 95–121. 19827947 10.1146/annurev-physiol-021909-135811 PMC2851227

[bib55] 55Verkman AS, Galietta LJ. Chloride channels as drug targets. Nat Rev Drug Discov 2009; 8: 153–171. 19153558 10.1038/nrd2780 PMC3601949

[bib56] 56Yousefi S, Gold JA, Andina N, Lee JJ, Kelly AM, Kozlowski E et al. Catapult-like release of mitochondrial DNA by eosinophils contributes to antibacterial defense. Nat Med 2008; 14: 949–953. 18690244 10.1038/nm.1855

[bib57] 57Fuchs TA, Abed U, Goosmann C, Hurwitz R, Schulze I, Wahn V et al. Novel cell death program leads to neutrophil extracellular traps. J Cell Biol 2007; 176: 231–241. 17210947 10.1083/jcb.200606027 PMC2063942

[bib58] 58West MR, Molloy CR. A microplate assay measuring chloride ion channel activity. Anal Biochem 1996; 241: 51–58. 8921165 10.1006/abio.1996.0377

