# Supplementary material for: Caspase-dependent cell death-associated release of nucleosome and damage-associated molecular patterns
Source: Cell Death Dis. 2014 Oct 30;5(10):e1494–. doi: 10.1038/cddis.2014.450 (PMC4649531; doi:10.1038/cddis.2014.450)
Supplement: Supplementary Figures [file cddis2014450x1.doc]

**
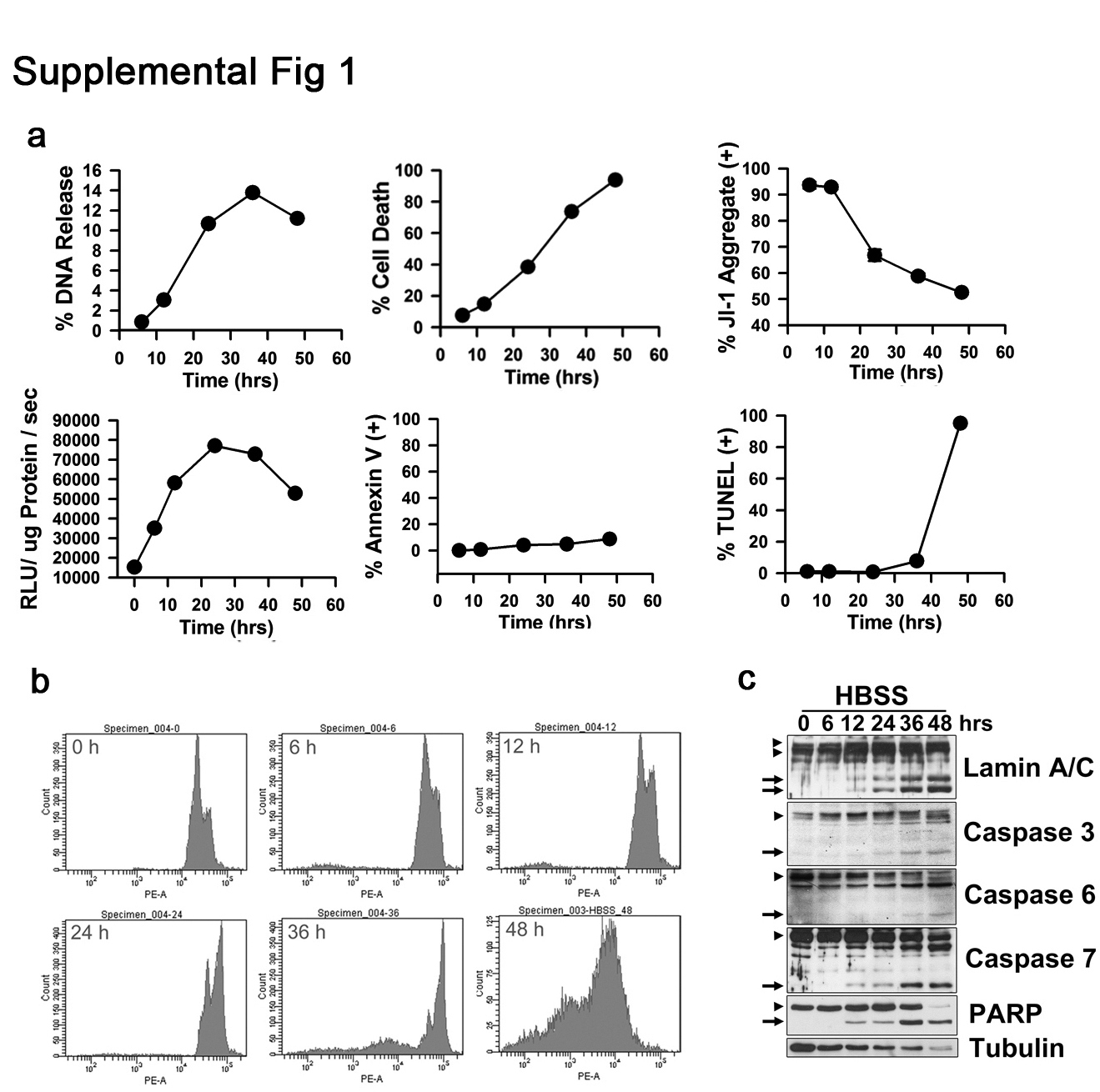
**

**Supplemental Figure 1. Apoptotic features of dying HeLa cells during amino acid deprivation.** (a) Various features of cell death from amino acid-deprived HeLa cells were measured by detecting damages of membrane integrities through propidium iodide staining (upper middle), TUNEL assay (lower right), Annexin V staining (lower middle), DNA release (upper right), measurements of mitochondrial membrane potential with JC-1 dye staining (upper right), luminescent assay for caspase 3/7 (lower left). Data are presented as mean ± SD. (b) SubG1 population of cellular DNA was measured by PI staining and flow-cytometric analysis in amino acid-deprived HeLa cells. (c) Degradation of effector caspases (3, 6, and 7) and caspase substrates (lamin A/C and PARP) was observed by Western blots (arrow heads: parental molecules; arrows: degraded fragments).

**
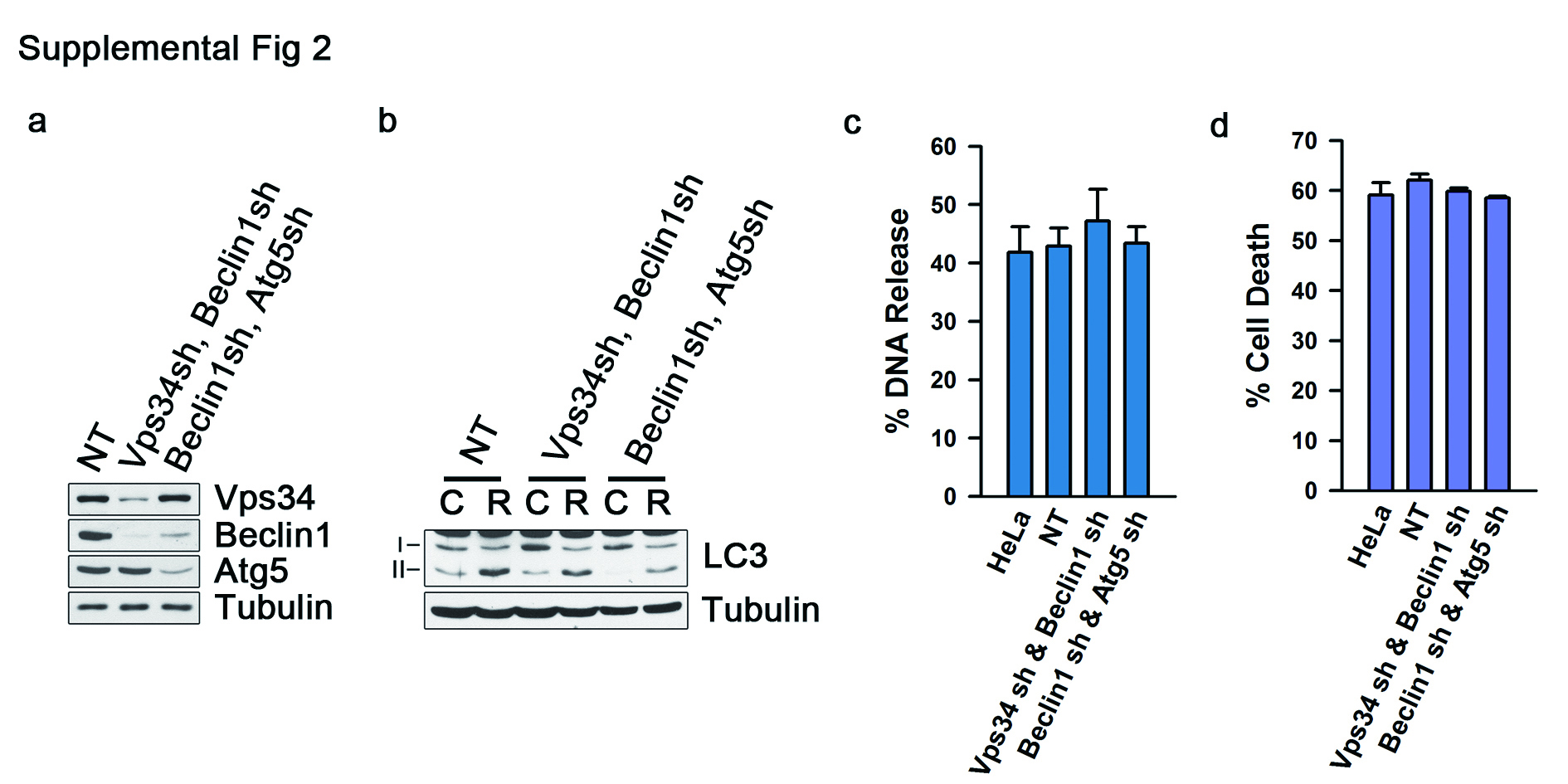
Supplemental Figure 2. Autophagy is not related to the release of nucleosomes and DAMPs in dying cells.** HeLa cells stably knocked-down with non-targeting (*NT*) shRNA, Vps34, and Beclin 1 shRNAs, or Beclin 1 and Atg 5 shRNAs were examined for expressions of Vps34, Beclin 1, and Atg5 by Western blots (a), successively, treated with control solvent (*C*) or 100 nM rapamycin (*R*) for 15 h, and Western blots for LC3 and tubulin were done for detecting autophagy (b). The cells were treated with staurosporine (1 μg/ml) for 10 h and released DNA was measured by PicoGreen dye staining (c) and fractions of dead cells were counted by PI staining and flow cytometry analysis (d). HeLa cells knocked-down for Vps34 and Beclin 1, or Beclin 1 and Atg5 showed no difference in either DNA release or cell death in comparison with controls notwithstanding reduced autophagy. Data are presented as mean ± SD (c, d).

**
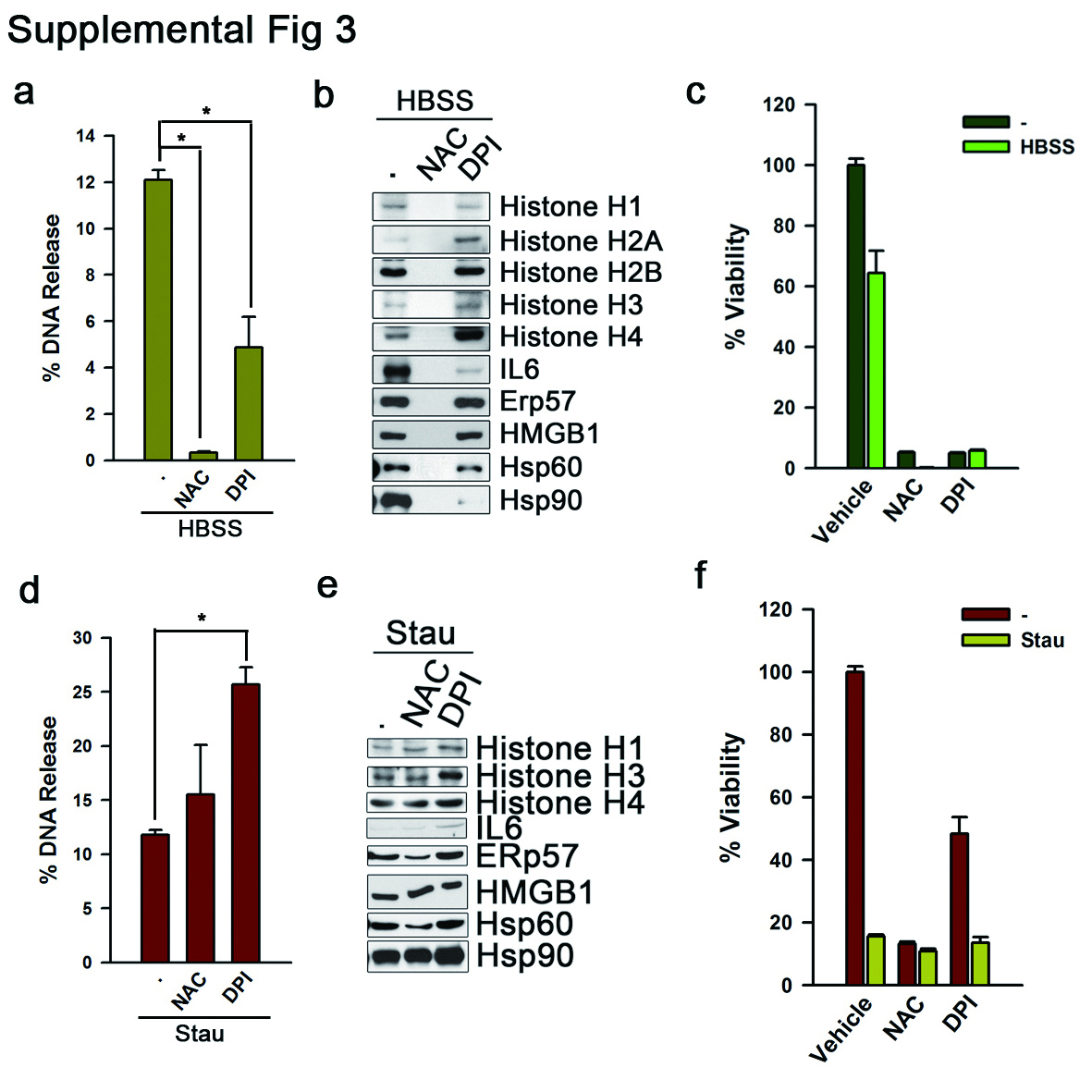
**

**Supplemental Figure 3. Release of nucleosomes and DAMPs is not dependent on reactive oxygen species.** HeLa cells were either incubated in amino acid-depleted medium for 24 h (HBSS) or treated with staurosporine (1 μg/ml) for 10 h in the presence or absence of anti-oxidants, 0.5 mg/ml N-acetyl-L-cysteine + 0.5 mg/ml ascorbic acid (NAC), or 50 μM diphenyliodonium (DPI). Released DNAs were measured by PicoGreen DNA staining (a, d) and released protein was Western-blotted for histones and DAMPs (IL6, ERp57, HMGB1, Hsp60, Hsp90) (b, e), and cellular viabilities were monitored by Calcein-AM assays (c, f). Anti-oxidants, N-acetylcysteine, and ascorbic acid or an inhibitor of Nox, diphenyliodonium, significantly ameliorated release of nucleosomes and DAMPs and cell viabilities in the amino acid deprived condition, whereas they substantially increased release of nucleosomes and DAMPs compared to control in staurosporine treatment. Therefore, ROS is not associated with nucleosomal and DAMPs release although ROS seems to be an associated factor with cell death induced by amino acids-deprivation. Data are presented as mean ± SD (a, c, d, and f). * P < 0.001

**
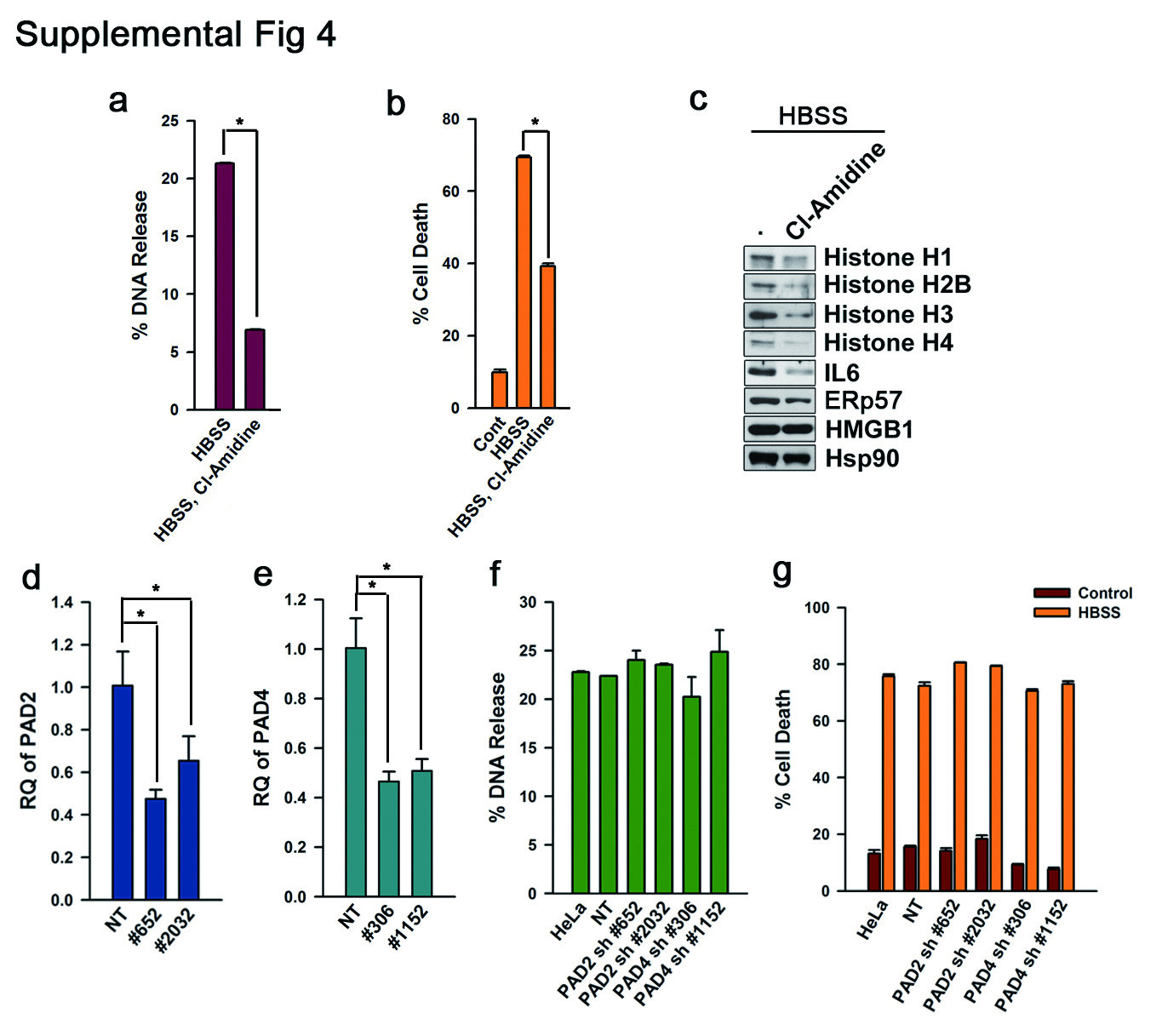
**

**Supplemental Figure 4. Effect of PAD inhibition on release of nucleosomes and DAMPs from dying cells.** HeLa cells were incubated in amino acid-depleted medium (HBSS) in the presence of PAD (peptidylarginine deaminase) inhibitor, Cl-amidine (20 μM) for 24 h. Released DNA and cell death were measured by PI staining (a) and flow cytometric analysis (b). Released proteins were examined by Western blots for histones and DAMPs (IL6, ERp57, HMGB1, Hsp90 (c). HeLa cells were transfected with non-targeting shRNA (NT), PAD2 shRNA (#652, #2032), or PAD4 shRNA (#306, #1152), whose expression of PAD mRNA was examined by real-time PCR (d, e). Amino acids were depleted through HBSS incubation for 24 h and released DNA was measured by PicoGreen staining and fluometric analysis (f). Cell death was checked by PI staining and flow cytometric analysis (g). Inhibition of cellular PADs by treatment of Cl-amidine reduced release of DNA, histones, IL6, and ERp5 as well as cell death, but knock-down of PAD2 or PAD4 showed no effect on DNA release and cell death. Therefore, arginine citrullination of histones by PAD appears not to be associated with nucleosomal and DAMPs release. Data are presented as mean ± SD (a, b, and d-g).* P < 0.001


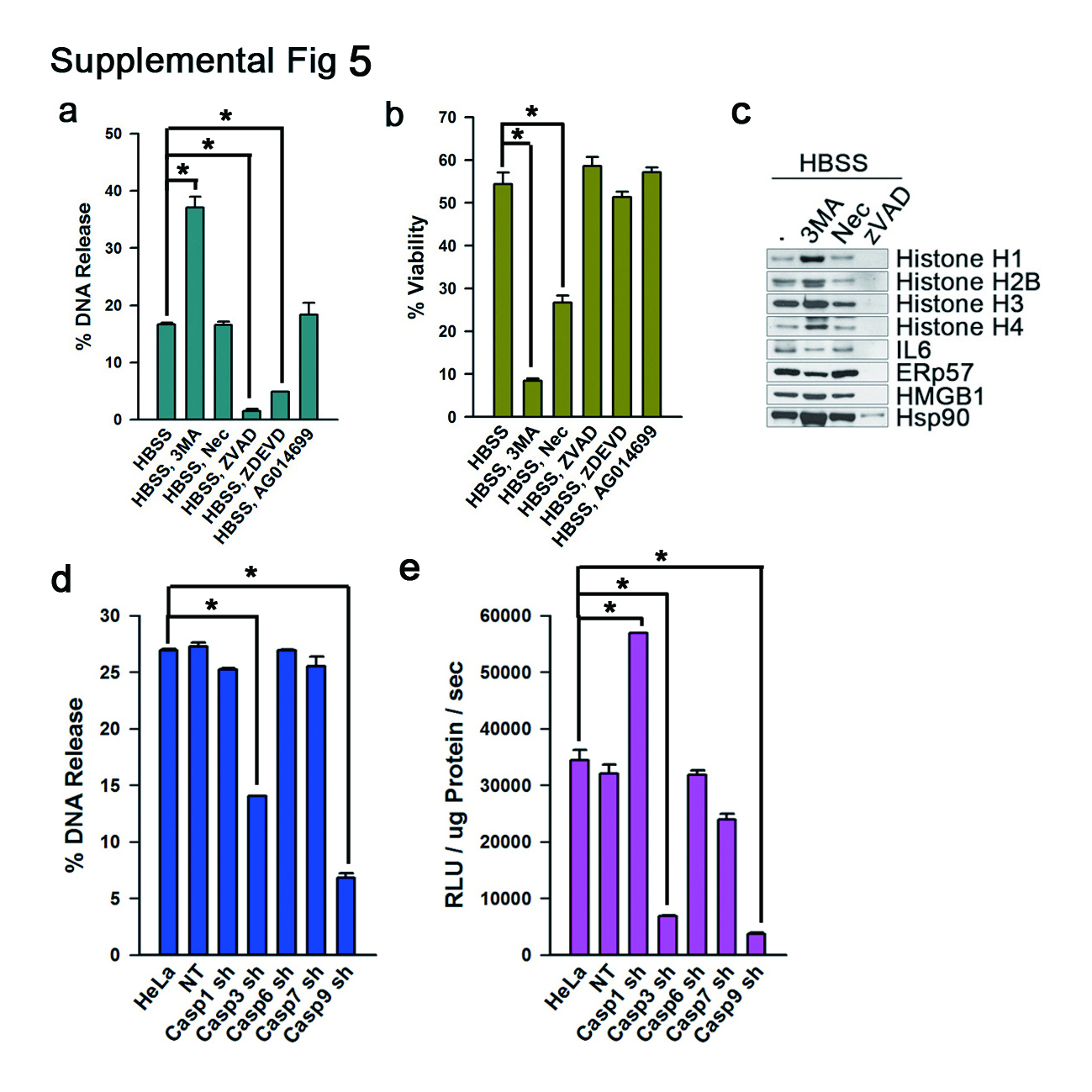


**Supplemental Figure 5. Nucleosome and DAMPs release from amino acid-depleted HeLa cells are dependent on caspase.** HeLa cells were amino acid-deprived by incubation with HBSS in the presence of 3-methylademine (3MA, 10 mM), necrostatin-1 (Nec, 20 μM), zVAD-fmk (20 μM), zDEVD-fmk (20 μM), or AG014699 (10 μM) for 15 h. Subsequently DNA release (a) and % viability (b) were measured by PicoGreen DNA dye and Calcein-AM assay, respectively. Released protein was Western-blotted for histones, IL6, ERp57, and Hsp90 (c). Parental HeLa cells or cells transfected with non-targeting shRNA (NT), caspase 1 shRNA, caspase 3 shRNA, caspase 6 shRNA, caspase 7 shRNA, or caspase 9 shRNA were incubated in amino acid-depleted medium (HBSS) for 15 h, from which the released DNAs were estimated by staining of PicoGreen DNA dye (d), and caspase 3/7 assay (e). Data are presented as mean ± SD (a, b, d, and e). * P < 0.001

**
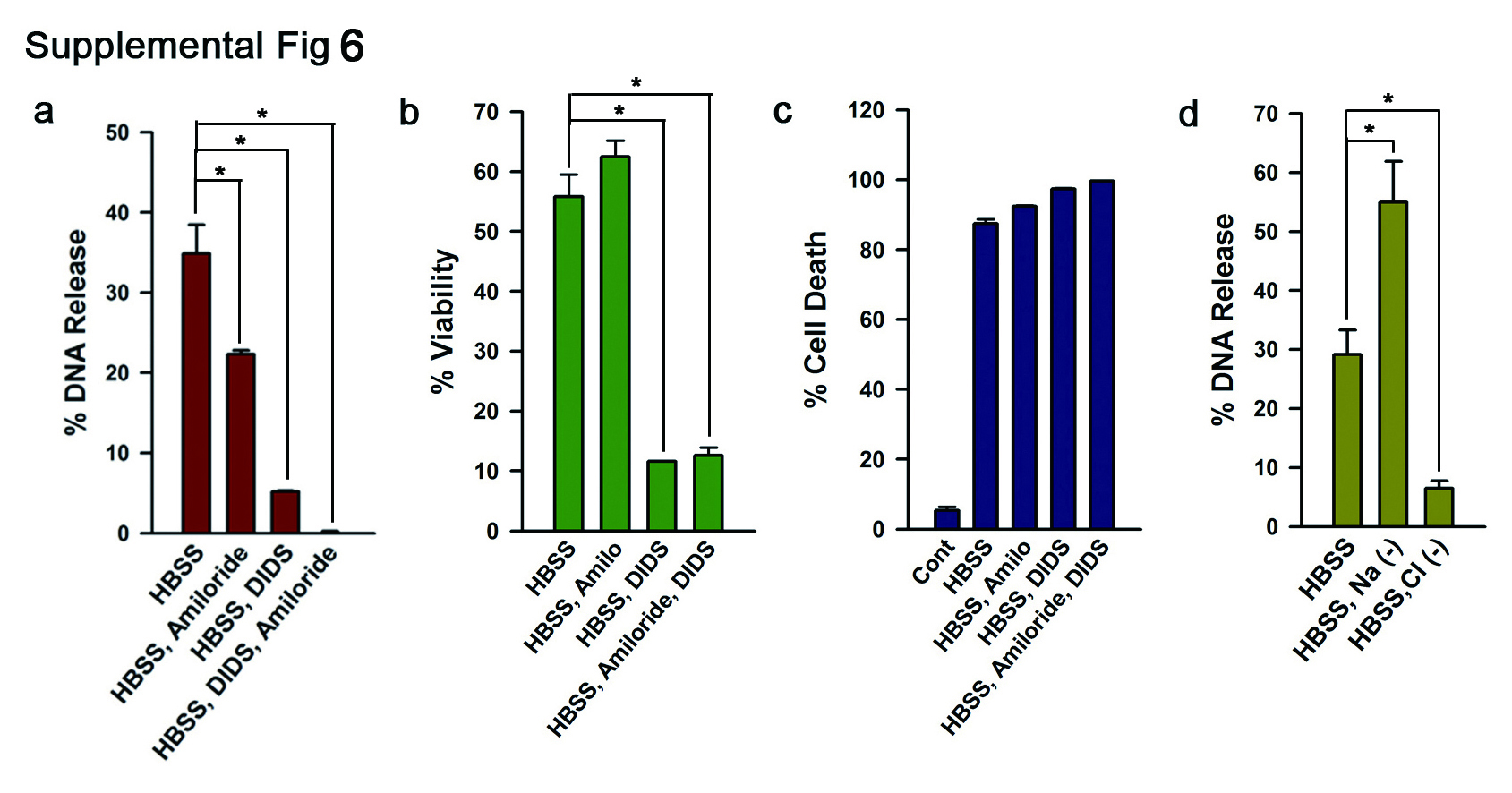
**

**Supplemental Figure 6.** HeLa cells were incubated in amino acid-depleted media (HBSS) in the presence of amiloride (400 μM), DIDS (200 μM), or amiloride and DIDS for 24 hrs. DNA release, cellular viability, or cell death were measured by PicoGreen staining, Calcein-AM assay, or SYTOX red staining, respectively (a, b, and c). HeLa cells were incubated in HBSS, Na^+^-deficient HBSS or Cl^-^-deficient HBSS for 24 hrs, and the released DNA was measured by PicoGreen method (d). Data are presented as mean ± SD. * P < 0.001.

**
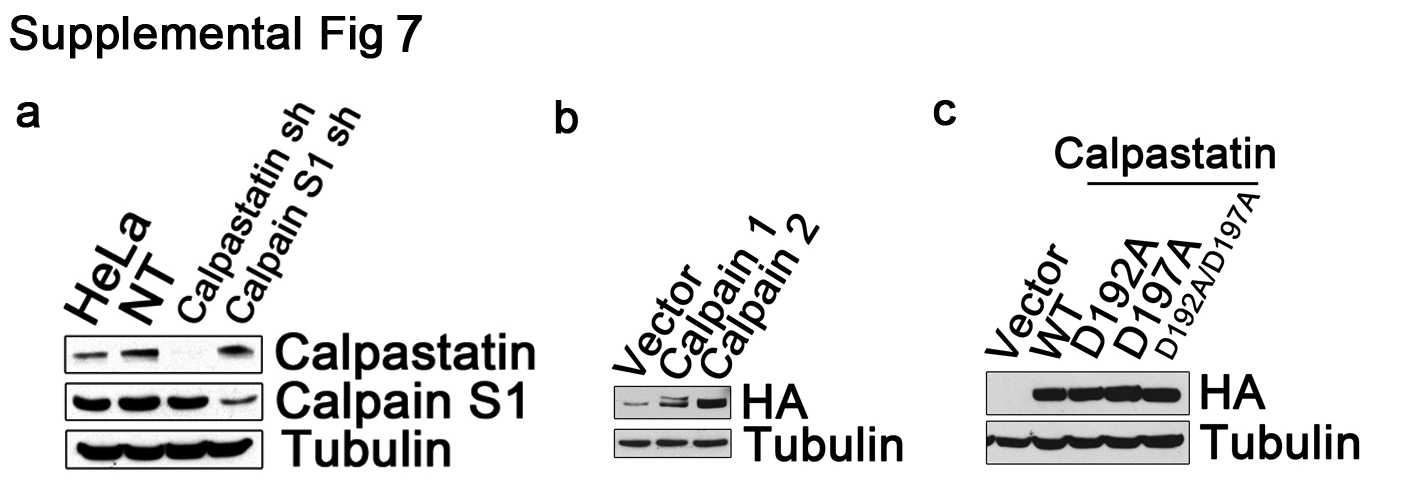
**

**Supplemental Figure 7.** (a) Expression of calpain S1 or calpastatin was tested by Western blots in HeLa cells transfected with calpastatin shRNA or calpain S1 shRNA. (b and c) HeLa cells transfected cDNAs of calpain 1, calpain 2, wild type calpastatin, or calpastatin mutants (D192A, D197A, and D192A/D197A) tagged with HA were tested for expressions of the constructs by Western blots.


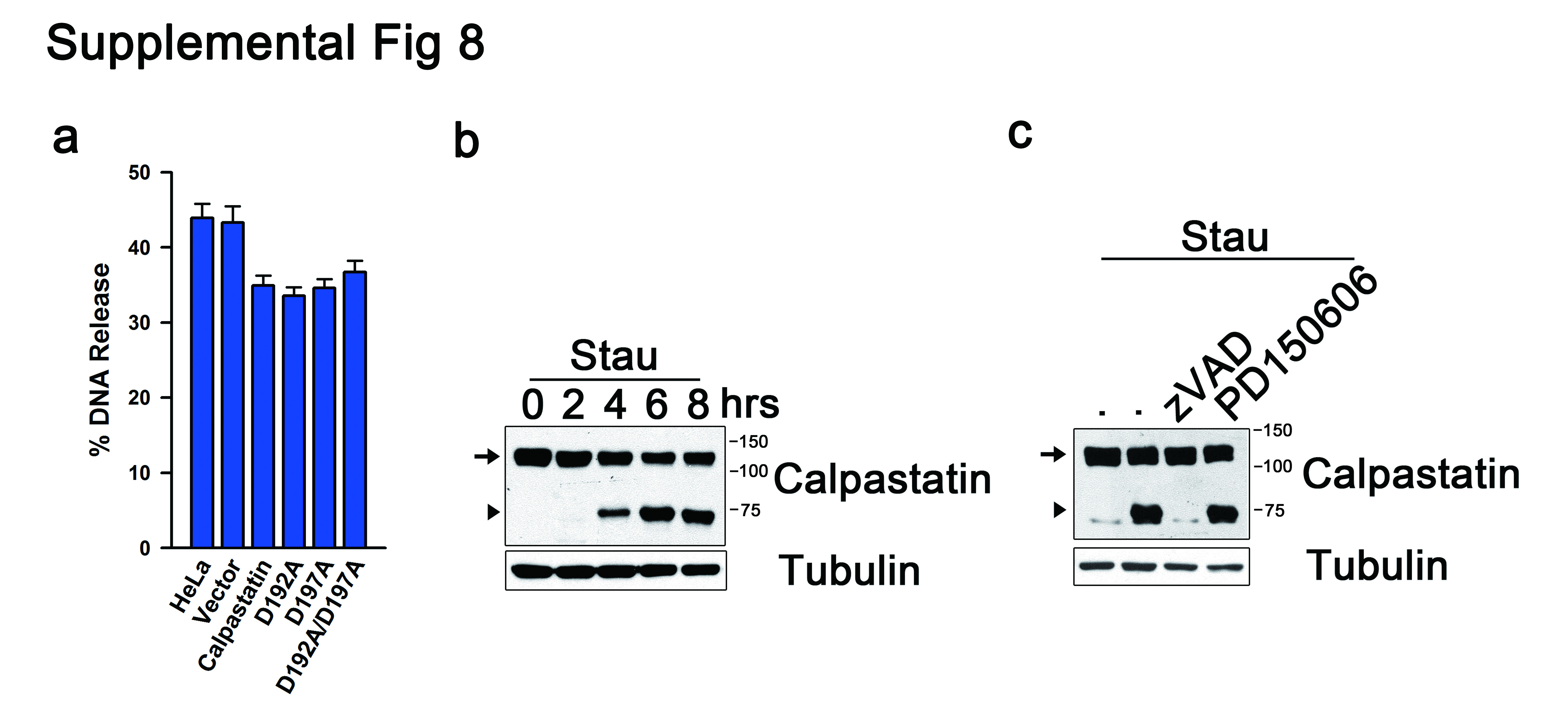


**Supplemental Figure 8. DNA release of dying cells is associated with calpain activity but not with calpastatin-mediated degradation of calpastatin.** Parental HeLa cells or cells transfected with control vector (*Vector*), wild type calpastatin cDNA (*Calpastatin*), mutant cDNAs of calpastatin at the sites of caspase-mediated cleavage (*D192A, D197A, D192A/D197A*) were incubated in staurosporine-containing media (1 μg/ml) for 10 hrs and the released DNAs were measured by PicoGreen method (a). Cells were treated with staurosporine for the indicated time periods (b) or for 6 h (c) with/without zVAD-fmk or PD150606 were Western-blotted for calpastatin (arrow, parental form; arrow head, cleaved form). Data are presented as mean ± SD (a).
